# Associations between genomic stratification of breast cancer and centrally reviewed tumour pathology in the METABRIC cohort

**DOI:** 10.1038/s41523-018-0056-8

**Published:** 2018-03-07

**Authors:** A. Mukherjee, R. Russell, Suet-Feung Chin, B. Liu, O. M. Rueda, H. R. Ali, G. Turashvili, B. Mahler-Araujo, I. O. Ellis, S. Aparicio, C. Caldas, E. Provenzano

**Affiliations:** 1 Department of Histopathology, Division of Cancer and Stem cells, School of Medicine, University of Nottingham, Nottingham, UK; 2 Nottingham University Hospitals NHS Trust, Nottingham, UK; 3 CRUK Cambridge Institute, Li Ka Shing Centre, University of Cambridge, Cambridge, UK; 4 Department of Pathology, University of Cambridge, Tennis Court Road, Cambridge, UK; 5 Department of Pathology and Laboratory Medicine, University of British Columbia, Vancouver, BC Canada; 6 Addenbrooke’s Hospital, Cambridge Breast Unit, Cambridge University Hospitals NHS Foundation Trust, Cambridge, UK; 7 NIHR Cambridge Biomedical Research Centre, University of Cambridge and Cambridge University Hospitals NHS Foundation Trust, Cambridge, UK

## Abstract

The integration of genomic and transcriptomic profiles of 2000 breast tumours from the METABRIC [Molecular Taxonomy of Breast Cancer International Consortium] cohort revealed ten subtypes, termed integrative clusters (IntClust/s), characterised by distinct genomic drivers. Central histopathology (*N* = 1643) review was undertaken to explore the relationship between these ten molecular subtypes and traditional clinicopathological features. IntClust subtypes were significantly associated with histological type, tumour grade, receptor status, and lymphocytic infiltration (*p* < 0.0001). Lymph node status and Nottingham Prognostic Index [NPI] categories were also significantly associated with IntClust subtype. IntClust 3 was enriched for tubular and lobular carcinomas, the latter largely accounting for the association with *CDH1* mutations in this cluster. Mucinous carcinomas were not present in IntClusts 5 or 10, but did not show an association with any of the remaining IntClusts. In contrast, medullary-like cancers were associated with IntClust 10 (15/26). Hormone receptor-positive tumours were scattered across all IntClusts. IntClust 5 was dominated by HER2 positivity (127/151), including both hormone receptor-positive (60/72) and hormone receptor-negative tumours (67/77). Triple-negative tumours comprised the majority of IntClust 10 (132/159) and around a quarter of IntClust 4 (52/217). Whilst the ten IntClust subtypes of breast cancer show characteristic patterns of association with traditional clinicopathological variables, no IntClust can be adequately identified by these variables alone. Hence, the addition of genomic stratification has the potential to enhance the biological relevance of the current clinical evaluation and facilitate genome-guided therapeutic strategies.

## Molecular taxonomy tracks pathology in breast cancer patient stratification

Molecular subtypes of breast cancer, defined by genetic alterations and expression patterns, tracks with traditional classification parameters. A team including histopathologists from the University of Cambridge, UK, and the University of Nottingham, UK, correlated the 10 subgroups of breast cancer previously discovered by analysing the genomic and transcriptomic architecture of 2000 tumours to standard diagnostic indicators routinely assessed by pathologists. Considering 1643 samples from these cases with available histopathological review data, the researchers showed that the novel subtypes were significantly associated with tumour type, tumour grade, receptor status, lymph node status, the extent of infiltration of anti-tumour immune cells, and the score of a commonly applied clinical assessment tool called the Nottingham Prognostic Index. However, these traditional variables alone could not fully demarcate the molecularly defined subgroups, suggesting that a combined approach holds potential value for evaluating patients and guiding therapeutic decision-making.

## Introduction

The molecular heterogeneity of breast cancer (BC) is well-recognised.^1–4^ This molecular diversity is currently poorly accounted for in the clinical setting. Approaches for effective and systematic genomic stratification of BCs are urgently required in order to facilitate therapeutic strategies.

The traditional classification of BC has utilised tumour morphology and assessment of oestrogen receptor [ER], progesterone receptor [PR] and human epidermal growth factor receptor 2 (HER2) expression. Expression signatures^1,3^ mostly reflect tumour classification based on these markers alongside proliferation, with luminal subtypes showing ER and/or PR expression, a HER2-positive subtype, and basal-like BCs being usually negative for all three receptors (triple-negative). This is now reflected in the most recent TNM prognostic stage grouping, which incorporates anatomic stage, grade, ER/ PR and HER2 receptor status and Oncotype Dx recurrence score [8th edition AJCC cancer staging manual].^5^

Next-generation sequencing has further refined the molecular profiles.^6,7^ The METABRIC (Molecular Taxonomy of Breast Cancer International Consortium) study identified ten subtypes of BC termed integrative clusters (IntClust), by joint analysis of copy number and expression data to detect the cis genomics.^8^ These ten subtypes show characteristic copy number aberrations (CNAs), and importantly are associated with distinct patterns of survival and response to neoadjuvant chemotherapy.^9^ IntClusts 3, 4, 7 and 8 have the best prognosis, IntClusts 1, 6 and 9 have an intermediate prognosis, and IntClusts 2, 5 and 10 a poor prognosis.^10^ IntClust 4 comprises a mixture of ER-positive and -negative tumours and is characterised by a relative paucity of CNAs and a gene expression signature reflecting immune activation. The majority of ER-positive and HER2-negative tumours are distributed within 8 IntClusts (1, 2, 3, 4, 6, 7, 8 and 9), but have variable degrees of genomic instability and distinct CNAs. For example, IntClust 3 has low genomic instability and a high frequency of *PIK3CA* mutations, IntClust 6 has amplification of 8p12 with upregulation of *ZNF703*, a common Luminal B BC oncogene^11^ and IntClust 2 has high genomic instability and amplification of 11q13/14. IntClust 10, composed of tumours with a high rate of *TP53* mutations and 5q deletion, has a very poor prognosis in the short term, but patients surviving beyond 6 years following treatment have an excellent long-term outcome. IntClust 5, associated with *HER2* amplification, has the worst prognosis in this cohort of patients derived from the pre-trastuzumab era.

The tumours in the original METABRIC cohort were collected between 1977–2005 from five centres in the UK and Canada. The original annotation of these tumours was based on the primary pathology reports, with obvious differences in terminology for the classification of histological tumour types over time and between the five contributing centres. Hence, the relationship between the IntClust subgroups and traditional clinicopathological factors has not been systematically investigated to date. Here, we have addressed this shortcoming by conducting detailed central review of the tumour pathology of the majority of cases comprising the original METABRIC study and have tested for associations with IntClust subtype.

## Results

### Patient profile

A total of 1643 cases (83%) from the METABRIC cohort were available for central pathology review. The key clinicopathological features of these cases are provided in Supplementary Table A (in Supplementary File 1).

### Histopathological parameters and IntClust associations

#### Tumour type

**Fig. 1:**
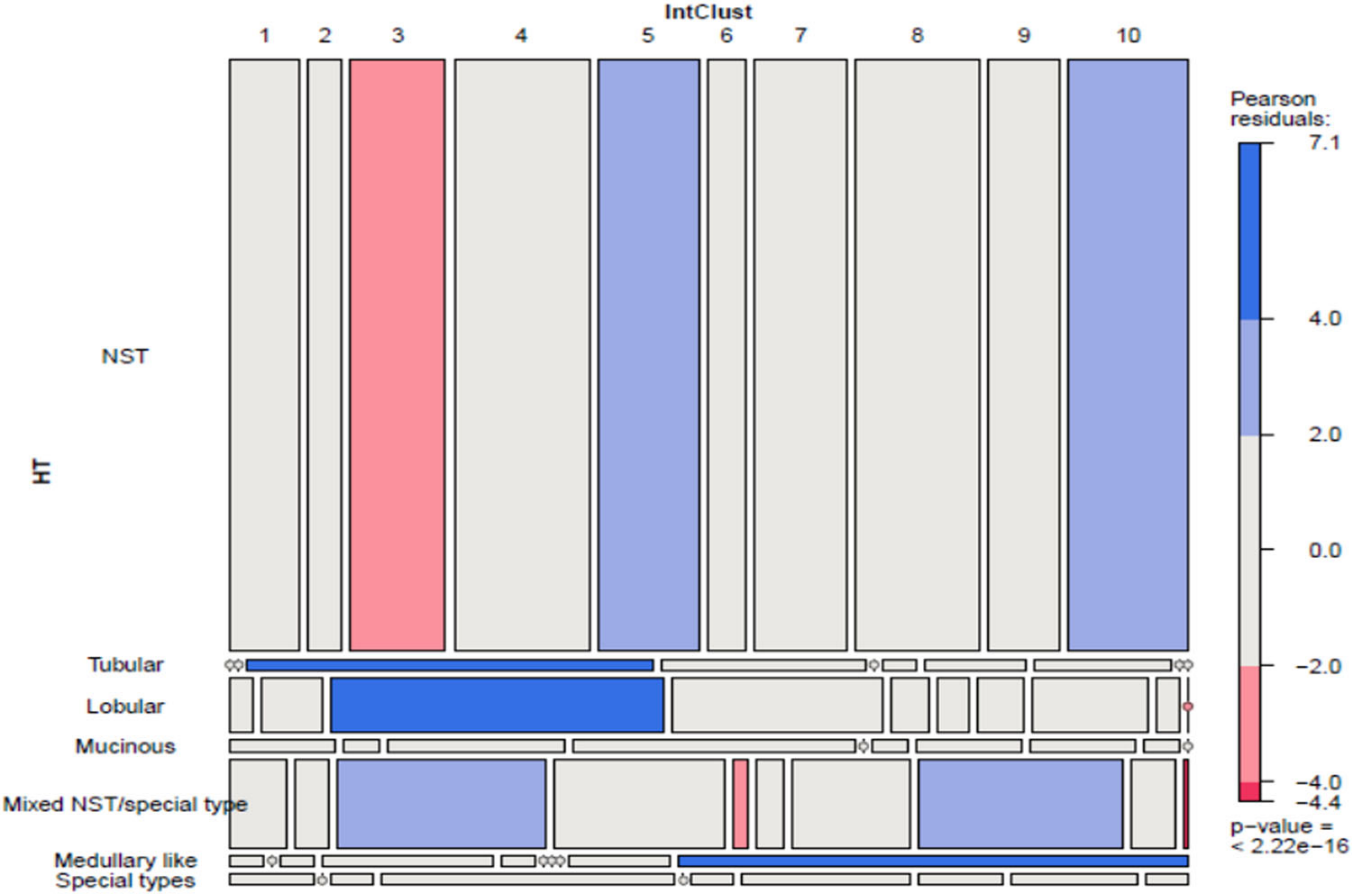
Integrative cluster associations with histopathological subtypes (HT) using Pearson Chi-square residuals

The IntClusts showed significant associations with tumour type (*p* < 0.000001; for significant Chi-square residuals see Fig 1; Table 1a for distribution). The commonest type, ductal NST, was distributed across all IntClusts; however, NST tumours were over-represented in IntClust 5 and 10, and under-represented in IntClust 3. IntClust 3 is particularly interesting, as it shows a strong association with tubular and lobular carcinomas, and an increase in mixed-NST/ special type tumours. IntClust 3 shows an association with *CDH1* mutations. On closer analysis, this is largely related to the lobular carcinomas within this IntClust; as would be expected, none of the tubular carcinomas in IntClust 3 harboured *CDH1* mutations (OR 0.38 for tubular vs. 20.42 for lobular BC). Mixed tumours were also increased in IntClust 8. Medullary carcinomas were associated with IntClust 10 (15/26; 58%), which did not contain any tubular, lobular or mucinous carcinomas. Mucinous carcinomas were distributed in IntClusts 3, 4, 7 and 8, but did not show an association with any one cluster.

**Table 1a:** Common breast cancer types vs. IntClusts

IntClust	Tubular carcinoma	Lobular carcinoma	Mucinous carcinoma	Medullary-like carcinoma	NST	NST-mixed	Other	Overall frequency of IntClust
1	0	3^a^	3^a^	1	98	12	2	119 (7.2%)
CSR	−1.4	−1.8	0.9	−0.6	0.8	−0.4	0.4	
2	0	8^a^	1^a^	0	49	7	0	65 (4%)
CSR	−1	1.6	0	−1	0	−0.1	−0.9	
3	12	43	5	1^b^	134	44	1	240 (14.6%)
CSR	4.2	6.5	0.7	−1.4	−3.5	3.2	−1.2	
4	6	27	8	5^b^	191	36	7	280 (17%)
CSR	0.7	1.7	1.8	−0.3	−1.5	0.8	1.8	
5	0	5^a^	0	1	142	3	0	151 (9.2%)
CSR	−1.5	−1.7	−1.5	−0.9	2.6	−3.4	−1.4	
6	1^a^	4^a^	1^a^	0	56	6	1	69(4.2%)
CSR	−0.1	−0.4	−0	−1	0.5	−0.6	0.1	
7	3	6	3	0	130	25	4	171 (10.4%)
CSR	0.2	−1.7	−0.2	−1.6	0	1.3	1.2	
8	4	15	3	0	176	43	2	243 (14.8%)
CSR	0.1	−0.5	−0.4	−2	−0.6	3	−0.6	
9	0	3^a^	1^a^	3	101	9	3	120 (7.3%)
CSR	−1.4	−1.8	−0.6	−0.8	1.1	−1.2	1.2	
10	0	0	0	15	168	1	1	185(11.3%)
CSR	−1.7	−3.6	−1.7	7.1	2.3	−4.4	−0.9	
Total	26 (1.6%)	114 (6.9%)	25 (1.5%)	26 (1.6%)	1245 (75.8%)	186 (11.3%)	21 (1.3%)	1643 (100%)

Frequency distribution of different pathological types of breast carcinomas across the ten integrative clusters (IntClust)

*CSR* Chi-square residual values

^a^Highlights location of traditionally good prognosis subtypes in the intermediate/poor prognostic categories

^b^Highlights distribution of a cohort of medullary carcinomas in the good prognosis clusters

Of 114 lobular carcinomas, 91 were in good prognosis IntClusts (80%), ten were in intermediate prognosis IntClusts (9%), and 13 were in poor prognosis IntClusts 2 and 5 (5% and 4%, respectively). 23 lobular carcinomas were of the solid/pleomorphic varieties, traditionally associated with worse clinical outcome. However, 13 of these fell in the good prognosis IntClusts (57%), and conversely, only 5/13 lobular carcinomas in IntClust 5 and 2 were of solid/pleomorphic subtype (Table 1b). While histologic subtype of lobular carcinoma did not explain the distribution across IntClusts, there was a significant association of grade 3 lobular cancers with worse prognosis IntClusts (*p* = 0.02). Looking at other variables, lesion size was not correlated with differential distribution of lobular BC across IntClusts (*p* = 0.46).

**Table 1b:** Special breast cancer subtypes vs. IntClusts

IntClust	NST with apocrine features	Solid/pleomorphic lobular	Invasive micropapillary	Invasive papillary with or without ductal NST	Adenoid cystic	Metaplastic	Pleomorphic carcinoma
1	1	2	0	2	0	0	0
2	0	3	0	0	0	0	0
3	2	8	1	0	0	0	0
4	5	3	1	2	2	1	1
5	3	2	0	0	0	0	0
6	2	1	0	1	0	0	0
7	1	1	1	3	0	0	0
8	3	2	0	2	0	0	0
9	5	1	2	1	0	0	0
10	9	0	0	0	0	1	0
Total	31	23	5	11	2	2	1

Distribution of some special types of cancers: solid/pleomorphic lobular; NST with apocrine features and the rare subtypes of BC

Ductal NST tumours with apocrine differentiation fell in all IntClusts except IntClust 2, with the largest proportion in IntClust 10 (9/31; 29%) (Table 1b). The distribution of the other rare BC subtypes is given in Table 1b.

#### Tumour grade

**Fig. 2:**
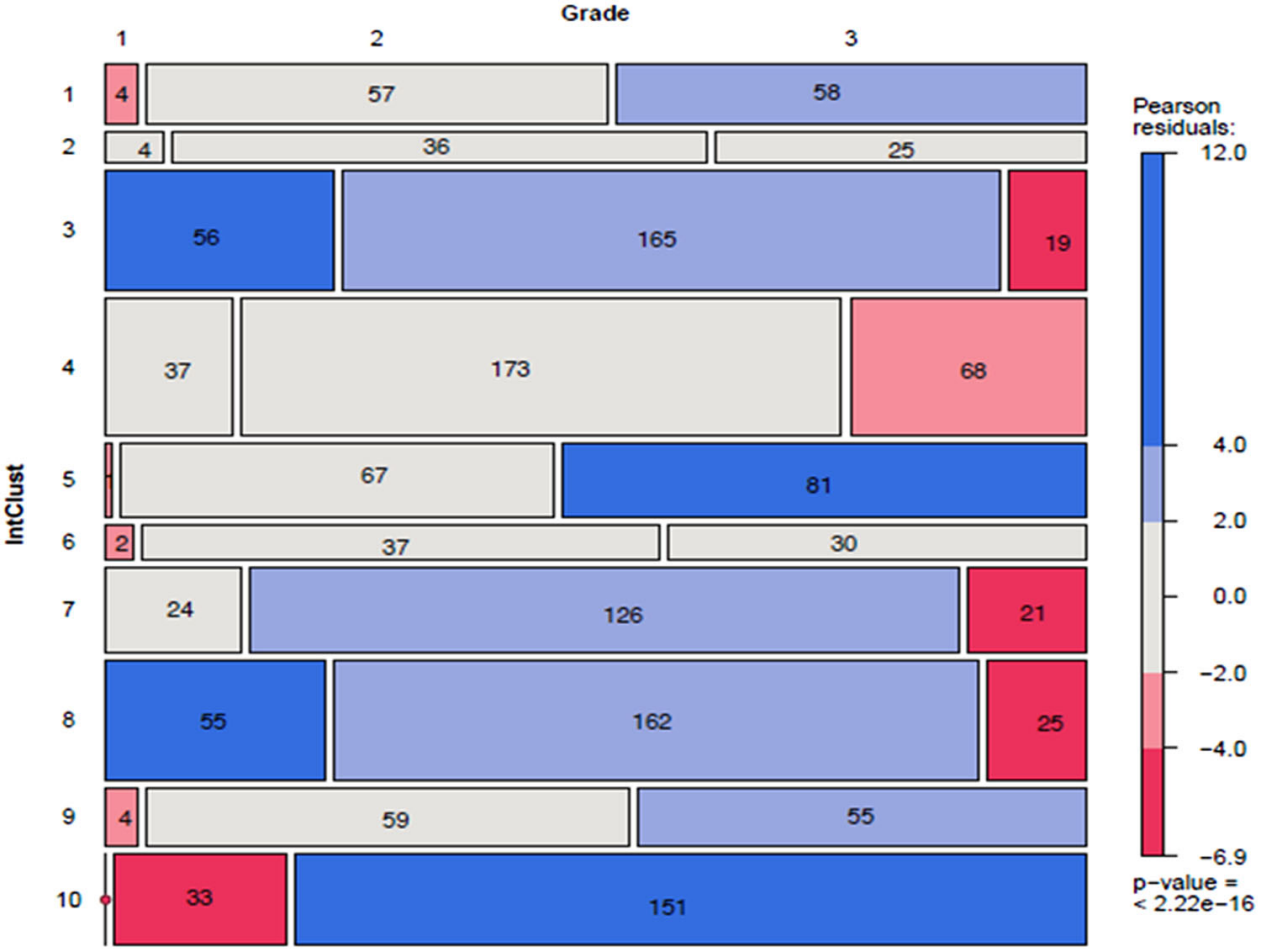
Pearson Chi-square residuals for Integrative Cluster (IntClust) associations with grade [distribution of grade 1–3 within IntClusts to be read left to right across *x*-axis; data labels show absolute values; areas within the tiles in the spine-plot are proportional representations; *x*-axis: (of the whole cohort); *y*-axis: (within each IntClust)]

IntClusts were significantly associated with the overall tumour grade (*p* < 0.000001; Fig. 2) and with each of the individual components (*p* < 0.000001) of grade: tubule formation, nuclear pleomorphism and mitotic count (Supplementary Table B in Supplementary File 1). Most grade 1 tumours fell in the good prognosis IntClusts (172/187; 92%. Fig. 2), and were positively associated with IntClusts 3 and 7 and negatively associated with 1, 5, 6, 9 and 10. Grade 2 tumours were spread across all IntClusts but showed significant associations with IntClusts 3, 7 and 8. Grade 3 tumours showed a positive association with intermediate and poor performing IntClusts 1, 5, 9 and 10, and a negative association with IntClust 3, 4, 7 and 8. Despite these associations, 25% of grade 3 tumours fell in the good prognosis IntClusts (133/533). IntClust 2 has a poor prognosis and is resistant to neoadjuvant chemotherapy, but showed no association with tumour grade.

The individual components of grade were examined separately. Tumours with high tubule formation (tubule score 1) predominantly fell in IntClusts 3, 4 and 8 (Suppl Table Bi.a), which also showed low mitotic counts (mitosis score 1) (Suppl Table Bii.a). Poor tubule formation (tubule score 3) was more evenly distributed across IntClusts. Tumours with high mitotic activity (mitosis score 3) were concentrated in IntClusts 5 and 10, and these IntClusts showed the highest proportion of tumours with pleomorphism score 3 (Suppl Table Biii.a). The overall distribution of grade 3 tumours mirrors the distribution of mitosis score more closely than tubule formation, however, grade 1 tumours reflect the distribution of both variables.

#### Lymphocytic infiltration

**Fig. 3:**
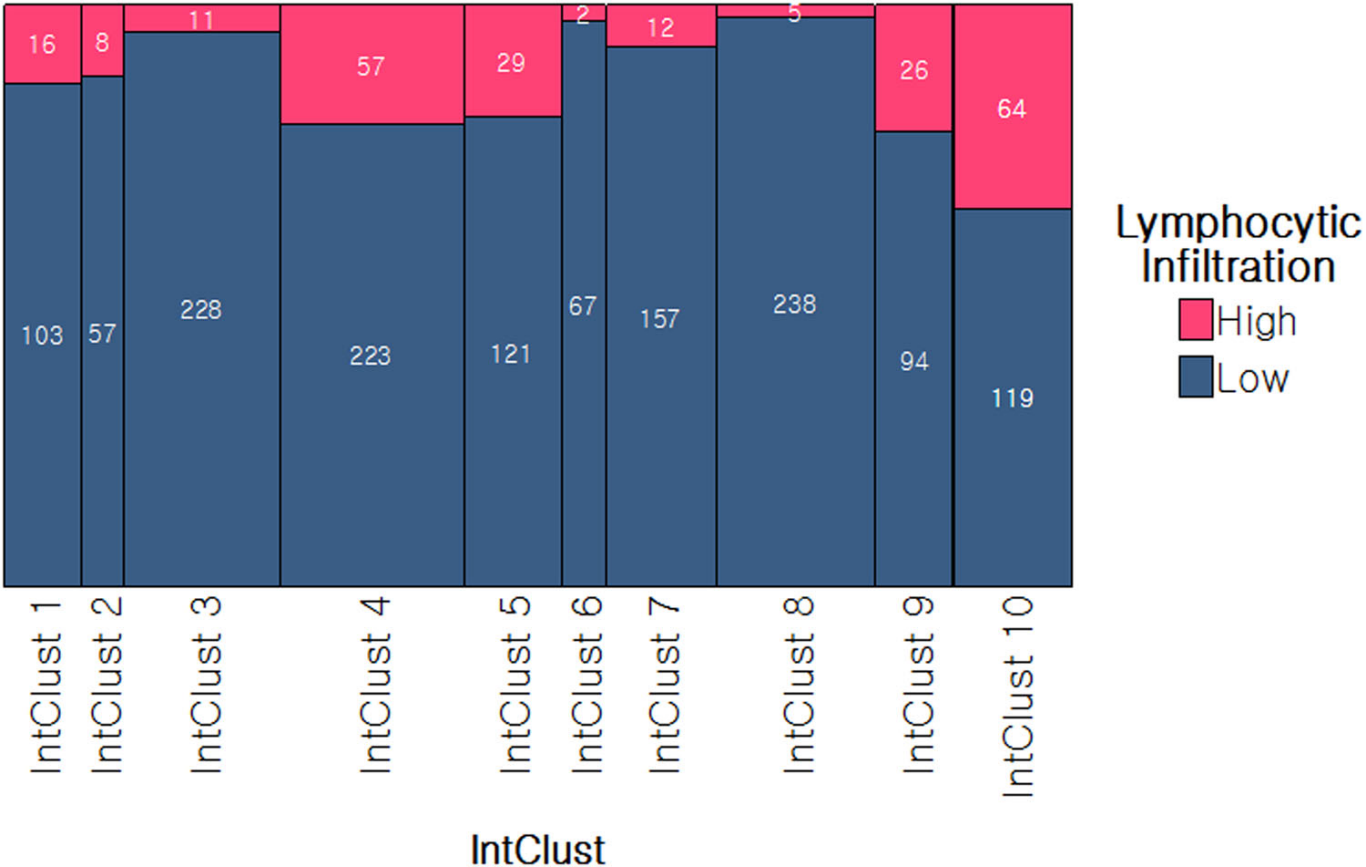
Lymphocyte distribution within Integrative Clusters (IntClust) [data labels show absolute values; areas within the tiles in the spine-plot are proportional representations; *x*-axis: (of the whole cohort); *y*-axis: (within each IntClust)]

There was a significant association between IntClusts and tumour lymphocytic infiltration (*p* < 0.001). Two-hundred and thirty of 1637 cases showed high levels of lymphocytic infiltration, and they were predominantly in IntClust 4 (*n* = 57) and 10 (*n* = 64) (Chi-square residuals 2.8 and 7.6, respectively), representing 20% and 35% of cancers in these IntClusts, respectively (Fig. 3).

#### Receptor status

**Fig. 4:**
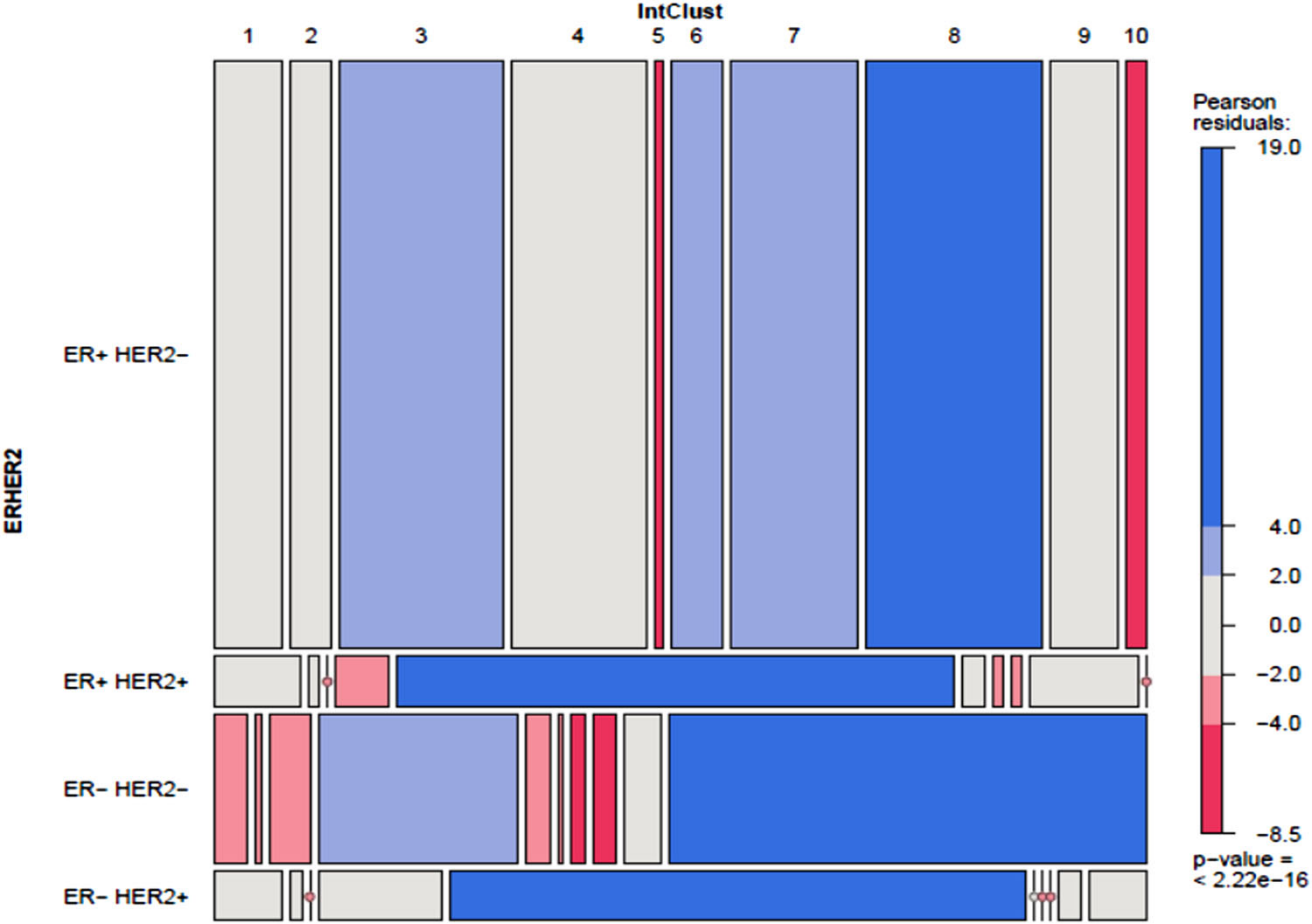
Pearson Chi-square residuals for IntCluster correlations with receptor subtype (ER/HER2)

Hormone receptor and HER2 status across the entire cohort is summarised in Supplementary Table 1 (in Supplementary File 1) and Supplementary Fig. 1. Data for all three receptors were available for 1321 cases (Supplementary Fig. 2) with significant associations observed between IntClust and combined receptor status (*p* < 0.0001). Strengths of association of ER and HER2 within IntClusts are depicted in the residual Chi-Square analysis in Fig. 4 (PR was excluded as it produced small subgroups limiting the statistical power).

ER + HER2- tumours were associated with IntClusts 3, 6, 7 and 8. ER + PR + cases formed the majority in IntClusts 2 (73%), 3 (64%), 6 (60%), 7 (69%), 8 (75%) and 9 (51%), while ER + PR- was the predominant fraction in poor prognosis IntClust 1 (47%).

HER2 status was available from IHC supplemented by copy number analysis where required for 1611 cases (Supplementary Fig. 1b). HER2 + tumours were associated with IntClust 5 regardless of hormone receptor status. The majority of HER2 + cancers clustered in IntClust 5 (127/198; 64%); conversely, 84% of cancers in IntClust 5 were HER2 + .

The majority of triple-negative cancers clustered in IntClust 10 (132/227; 58%) and formed the largest proportion of cases within this group (132/159; 83%); of the remainder, 23% (52/227) group in IntClust 4 and form 24% of this cluster (52/217).The remaining IntClusts except IntClust 5 showed a negative association with ER−HER2- status.

#### Lymph node-positive status and Nottingham Prognostic Index [NPI]

**Fig. 5:**
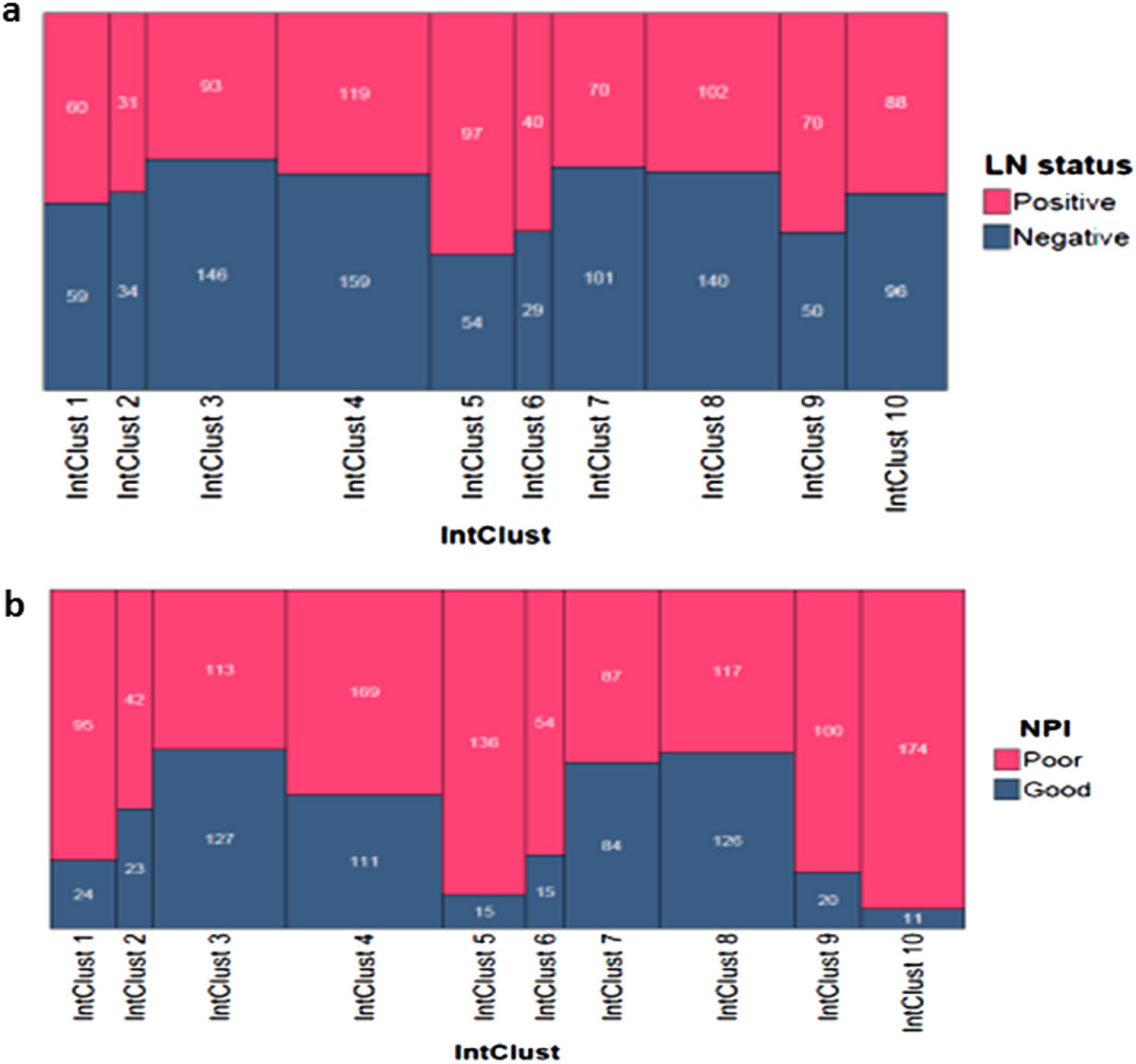
Prognostic features: **a** Lymph node positivity and **b** NPI categories vs. Integrative Clusters (IntClust) [data labels show absolute values; areas within the tiles in the spine-plot are proportional representations; *x*-axis: (of the whole cohort); *y*-axis: (within each IntClust)]

A significant association was observed between lymph node status and IntClust (*p* < 0.001). IntClusts 1, 5, 6 and 9 showed a greater frequency of lymph node positivity compared to other IntClusts (Fig. 5a; 50%, 64%, 58% and 58%, respectively) with the strongest association in IntClust 5. IntClusts 3, 4, 7 and 8 showed the lowest rates of lymph node positivity (39%, 43%, 41% and 42%, respectively). In the lymph node-positive group (*n* = 770), 384 cases (49%) were in good prognosis IntClusts, and only 216 (28%) were in the poor prognosis IntClusts. Lymph node negative cases were mostly (546/868, 63%) distributed in good prognosis IntClusts, but 138 (16%) were in intermediate prognosis and 184 (21%) were in poor prognosis IntClusts.

Given the strong associations with grade and lymph node status, it is not surprising that IntClust stratification was also significantly correlated (*p* < 0.001) with the NPI status (Fig. 5b). Tumours in the poor prognosis IntClusts 5 and 10 had mostly high NPI (poor and very poor NPI categories combined), 90% and 94%, respectively, (Chi-square residuals 3.6 and 4.7, respectively). But high NPI was also present in intermediate (IntClust 1–80%, IntClust 6–78% and IntClust 9–83%) and good prognosis IntClusts (IntClust 3–47%, IntClust 4–60%, 51% IntClust 7–51% and IntClust 8–48%).

#### Subgroup analysis of IntClust based on ER status

All IntClusts were analysed for subgroups based on ER status, with distinct subgroups observed within IntClust 4. Few significant observations were made in other IntClust, which were either predominantly ER + or ER−.

##### IntClust 4

Seventy-five cases were ER − (27%) and 204 were ER + (73%); ER status was unavailable in 1 case. The ER + tumours were of lower grade (17% grade 1 and 15% grade 3), compared with ER − tumours (50% grade 3 and 4% grade 1; *p* < 0.0001). Heavier lymphocytic infiltration was observed in ER − (43%) vs. ER + cancers (12%) (*p* < 0.001). ER + cancers were evenly spread across good and poor NPI categories (46% and 54%, respectively), whereas ER − cancers were skewed towards poorer NPI prognostic categories (79%) (*p* = 0.0004).

#### Prediction of IntClusts from clinicopathological variables: regression models

There is only a 39% agreement between IntClusts predicted from clinicopathological variables vis a vis DNA/RNA classifier (Supplementary Fig. 3a). The probability of prediction of each individual IntClust based on clinicopathological variables alone was < 0.25 for all IntClusts (Supplementary Fig. 3b). A model of conditional independence including grade or receptor status did not explain the distribution of histological types within each IntClust (Supplementary Table Ci and Cii in Supplementary File 1).

#### Comparison of IntClust classification vs. other classifiers

The predominant PAM50 category distribution among the IntClusts was as follows: 1 (LumB 66%); 2 (LumB 48%); 3 (LumA 69%); 4 (LumA 34%); 5 (Her2 55%); 6 (LumB 49%); 7 (LumA 66%); 8 (LumA 67%); 9 (LumB 48%) and 10 (Basal-Like 88%) (Supplementary Table Di in Supplementary File 1).

The predominant SCMGENE^12^ category distributions among the IntClusts were as follows: ER + /Her2-ve, high proliferation BCs formed the predominant subtype in IntClusts 1 (63%), 2 (66%), 6 (71%), 9 (67%) while ER + /Her2-ve, low proliferation BCs predominated in IntClusts 3 (70%), 4 (45%), 7 (46%) and 8 (51%). Eighty-three percent of IntClust 5 was Her2 + while 75% of IntClust 10 was ER − /HER2-ve (Supplementary Table Dii in Supplementary File 1). All currently known genomic and pathological associations for IntClusts are summarised in Supplementary Table E.

## Discussion

Following central review of tumour pathology, we found that the genome driver-based IntClust subtypes of BC show characteristic patterns of association with clinicopathological variables. Our findings highlight that molecular BC subtyping based on multi-platform analyses converge with traditional histopathological features, and that the two are complementary.

Our study has several limitations. Materials were from archival samples at multiple institutions and pre-dated the trastuzumab era. There is a bias towards banking of large cancers in the historical tumour bank collections that comprise the METABRIC cohort (as compared to 70% T1 tumours in ONCOPOOL,^13^ only 43% were T1 in METABRIC) and this may skew the distribution of pathological and prognostic variables within the IntClusts. Histopathological parameter modelling of prognostic effects within the IntClusts, though attempted, was underpowered in sub-categories to draw definite conclusions. Addressing the prognostic and predictive effects of histological features within the IntClusts will require analyses on larger data sets from multiple sources, with detailed pathological and clinical annotation.

Tubular carcinomas, a well-recognised special type of BC, showed the least variability and were distributed in the good prognosis IntClusts consistent with the excellent behaviour of tubular carcinomas documented in other series.^14^ The strongest association was with IntClust 3, defined by low genomic instability and a high incidence of *PIK3CA* mutations. Of the remaining tubular carcinomas, 3 fell in IntClust 7 (with 16q loss and *MAP3K1* mutations), 4 fell in IntClust 8 (1q gain, 16q loss and *PIK3CA* and *GATA3* mutations) and 6 belonged to IntClust 4 (few copy number alterations and activation of immune pathways). This illustrates that a histologically distinct special type BC can have diverse genomic drivers.

Other typically ER + histological types, including lobular and mucinous cancers, showed a more variable distribution across IntClusts. IntClust 3 is enriched for *CDH1* mutations, and this is secondary to the association with lobular carcinoma. Although the majority of lobular carcinomas belong to the good prognosis IntClusts, a significant number were found in the intermediate and poor prognosis IntClusts. This distribution was not explained by pleomorphic or solid subtype, but showed an association with grade 3 lobular tumours. This supports the findings of Rakha et al.,^15^ who found an association with worse prognosis for grade 3 lobular carcinomas, but not the pleomorphic subtype on its own. Of interest, the two largest groups of lobular carcinomas were present in IntClust 3 and 4; both defined by low genomic instability. The former is associated with frequent *PIK3CA* mutations, while the latter is associated with immune response. Immune related and hormone-related subtypes of lobular carcinomas have been recently described in the TCGA (The Cancer Genome Atlas)^16^ and RATHER cohorts.^17^ This may offer novel treatment options for lobular BC dependent upon the molecular profile.

Mucinous tumours were relatively evenly distributed across all IntClusts except IntClusts 5 and 10. This finding highlights the heterogeneity in underlying genomic drivers of mucinous tumours despite their distinctive morphological appearance and typical ER + HER2− phenotype. Mucinous carcinomas less frequently harboured 1q and 16p gain and 16q and 22q loss compared to other histotypes in this series, as described by others.^18^

The prognostic relevance of medullary-like BC remains controversial.^19^ Here, most triple-negative and basal-like cancers including medullary-like tumours belonged to IntClust 10, which is associated with the greatest genetic instability, harbouring characteristic *cis*-acting alterations (5q loss and 8q, 10p and 12p gain) associated with impaired DNA damage repair and cell-cycle checkpoint regulation. However, a significant proportion also fell in IntClust 4 characterised by low genomic instability and prominent lymphocytic infiltration. A CD8 + lymphocyte infiltrate is a good prognostic feature in basal-like cancers^20,21^ and further evidence is emerging that lymphocytic infiltration may risk stratify patients.^22^ It has become apparent that a subset of ER − breast tumours, including those that belong to IntClusts 4 and 10, are associated with a significant immune response conferring better outcome.

Some special type tumours were present in relatively small numbers in the series precluding ability to draw definitive conclusions. Papillary carcinomas, which are a mixture of Luminal A and B types,^23^ fell within good and intermediate prognosis IntClusts. Despite their association with lymphovascular invasion and suggested poorer outcome in some series,^24^ micropapillary carcinomas fell across the good prognosis IntClusts 3, 4 and 7 with two in IntClust 9 defined by high-genetic instability, 20q amplification and intermediate prognosis. The two Adenoid cystic carcinomas both fell into IntClust 4, consistent with their known indolent behaviour despite their basal-like features, and have recently been shown to harbour a characteristic *MYB-NFIB* gene translocation with low background genetic instability.^25,26^

Ductal NST carcinomas lacking the characteristics of a special histological type form a morphologically and prognostically heterogeneous group, so it is unsurprising that they are distributed across all IntClusts. Two thirds of the NST carcinomas showing apocrine differentiation fell in the intermediate and poor IntClusts, with only 10% in IntClust 5 consistent with reported HER2 positivity rates for these tumours.^27^ Interestingly, mixed-NST and special type tumours predominantly fell in the good prognosis ER + IntClusts 3 and 8, possibly reflecting more indolent underlying molecular biology related to the special type component.

Grade is a routinely assessed traditional pathological prognostic factor, which shows strong association with patient outcome.^28^ Although there was an association between grade and IntClust, tumours of grades 1–3 fell across all IntClusts except 10. Grade 1 tumours were more predictive of IntClust subtype, whereas grade 3 tumours were found in all IntClusts including those associated with good clinical outcome. Hence, tumour grade has limited utility in predicting the underlying genomic drivers of an individual cancer. Grade 3 cancers in intermediate and poorer prognostic IntClusts had a higher proportion of BC-specific deaths [1: 31%; 2: 44%; 5: 38%; 6: 30%; 9: 27%; 10: 29%] than grade 3 cancers in good prognosis IntClusts [3: 16%; 4: 21%; 7: 10%; 8: 24%]. Hence the genomic heterogeneity may explain differential rates of BC-specific death within grade 3 BC.

Looking at the individual components of grade, poor tubular differentiation was the only component that was significantly associated with BC-specific deaths in the whole cohort (*p* = 0.001), as well as ER + (*p* = 0.02) but not ER-ve subgroups (*p* = 0.89). A recent study of the molecular portraits of BC including the METABRIC cohort^29^ indirectly surmised that differentially expressed genes associated with poor tubular differentiation differed from signatures of either marked nuclear pleomorphism or higher mitotic counts, which were both enriched for proliferation genes. Given the sporadic distribution across IntClusts for BCs with poor tubular differentiation, it is unsurprising that these are not enriched for a particular gene-set, and the tubular differentiation component of grade may be a product of several biologically complex events.

Interestingly in METABRIC the genomic diversity of ER + tumours seems to be more prominent than that of ER − tumours. A proportion of every IntClust was formed by ER + tumours. This mirrors the findings of Horlings et al.^30^ who observed that ER + BCs are characterised by considerable variation in levels of genetic instability.

HER2 + tumours predominate in IntClust 5 regardless of ER status, but they also occur in other groups. The diversity of clinically HER2 + tumours has been reported previously; using PAM50 only 64% are assigned to the HER2-enriched group, the remainder distributing across all intrinsic subtype categories.^31,32^ Clinical HER2 status was shown to be non-contributory to prognosis after intrinsic subtype categorisation.^31^ Our findings provide a further example of the added value of molecular tumour subtyping in identifying tumours where HER2 is the primary molecular driver regardless of clinical HER2 status. There were 64 cases where there was a discrepancy between HER2 immunohistochemistry performed on Tissue Micro-Arrays (TMAs) and *HER2* copy number data (HER2 positivity defined as copy number > 6). Of these, 59 cases (92%) appear to have false-negative immunohistochemical staining, with a *HER2* copy number ranging from 6.02 to 51.2 (mean 12.9). False-negative immunohistochemistry results may be explained by inadequate fixation in this historical series of tumours, and by sampling errors in TMAs related to intralesional heterogeneity. Fourteen false-negative cases fell within IntClust 5 (mean *HER2* copy number 22.4), strengthening the association between this IntClust and *HER2* amplification. An analysis of the NOAH study found that HER2-enriched tumours showed a higher pathological complete response rate following trastuzumab therapy compared with non-HER2-enriched clinically HER2 + tumours.^33^ Patients in the METABRIC cohort had not received trastuzumab, and it remains to be demonstrated if the IntClust 5 group respond better to HER2-targeted agents.

Pathway analysis within the IntClusts can help us understand why some tumours with distinctive morphology behave aberrantly in terms of prognosis. Molecular interrogation of different histological subtypes of BC for such new drivers will help further unravel links with microscopy. For example, the molecular profile of ER-positive luminal A BCs has been investigated from six different data sets including the TCGA and METABRIC.^34^ The copy number high Luminal A sub-class has been shown to have a poorer prognosis compared to the low copy number variety.^34^ This category harbours 8q gain, 5q loss and 20q gain and parallels the intermediate and poor prognosis IntClusts in the METABRIC landscape. Given the ability of METABRIC IntClusts to provide prognostic information, it is unsurprising that the IntClusts associate strongly with parameters such as grade and lymph node status and to the traditional NPI.^35^ However, positive lymph node status or high NPI can occur even in the good prognosis IntClusts. IntClust subtype provides a basis for the behavioural diversity in historical prognostic models, and integration of genomic data with clinicopathological factors offers superior prognostic models to either variable alone.^36,37^

In summary, we have conducted a central pathology review of the largest case series of BC with matched genomic data. While the genome-driven IntClusts are significantly associated with traditional clinicopathological variables, IntClust subtype could not be reliably predicted based on current histological parameters alone. Adoption of genome-guided therapeutic strategies first requires classification of tumours into homogenous groups in terms of genomic alterations. IntClust subtypes are classified on this basis; therefore, a test for prospective assignment of IntClust subtype in the clinical setting, especially for predictive analyses, would be useful.

## Methods

### Genomic and transcriptomic profiling and generation of integrative clusters

All patient samples were acquired with appropriate consent from respective institutional review boards (REC ref 07/H0308/161; REC ref 12/EE/0484; REC ref 07/Q0106/63). DNA and RNA isolation from samples was followed by hybridisation to the Affymetrix SNP 6.0 and Illumina HT-12 v3 platforms for profiling. Full details of genomic and transcriptomic profiling and CNA/ variation analyses and allocation to integrative clusters are described in Curtis et al.^8^ IntClusts 3, 4, 7 and 8 are defined as the good prognosis groups, IntClust 1, 6 and 9 as intermediate prognosis groups, and IntClust 2, 5 and 10 as poor prognosis groups.^10^

### Inclusion criteria and histopathology review

Cases were included from the METABRIC cohort (*n* = 1980) only if histology slides were available for central pathology review along with matched clinical and expression analyses data. Any cases with no tumour slides available or non-invasive disease only were excluded from analysis.

A representative H&E section from each case was reviewed by a specialist breast pathologist (A.M., E.P., I.O.E., G.T., B.M.-A.). The following variables were assessed: histological tumour type,^38^ histological grade^39^ (including the individual components tubule formation, nuclear pleomorphism and mitotic frequency), lymphocytic infiltration, presence of lymphovascular invasion, and presence of in situ disease (DCIS and LCIS) and precursor lesions. For purposes of analyses, tumour types were grouped as follows: tubular (including tubular, cribriform and tubulo-lobular cancers), lobular (including classical, alveolar, solid and pleomorphic patterns), mucinous, medullary-like, NST, mixed-NST and special type, and other rare types (including metaplastic, adenoid cystic, papillary, micropapillary, apocrine).

At least 25% of the cases (*n* = 405) were reviewed by two or more of the study histopathologists. Any discrepancies in histological type and grade between the central reviewers were resolved by consensus review of scanned images. The concordance between the specialist breast pathologists was excellent (kappa statistic 0.9) for all variables assessed.

Information on lymph node stage and tumour size was available from original histopathology reports. Lymphocytic infiltration was categorised as either ‘low’ (absent to mild) or ‘high’ (moderate to severe).

### Receptor status determination

Tissue micro-arrays were constructed from the BC cases as per standard protocol.^40^ Micro-arrays were stained for ER, PR and HER2 using routine immunohistochemistry protocols summarised in Supplementary Table F (Supplementary File 1).^41^ The proportion of cells staining positively (Allred score) was used to determine ER and PR status: 0 = 0, < 1% = 1, 1%–9% = 2, 10%–32% = 3, 33–66% = 4, > 66% = 5.^42^ Any score of 2 or above was deemed positive. For cases with missing IHC data (due to missing/unavailable cores), the ER, PR status was supplemented from the histopathology reports at the time of diagnosis. HER2 was scored as per UK guidelines.^43^ A 2 + score or missing HER2 status was supplemented by results from original histopathology reports, if available, or from METABRIC HER2 gene copy number analysis ( > 6.0 copies deemed positive).^44^

### Prediction of IntClusts from clinicopathological variables: regression models

In order to determine whether clinicopathological variables could predict molecular subtype, ten multivariable logistic regression models were fit for each of the ten IntClust groups, with the dependent variable as one IntClust vs. the remaining nine clusters. Clinical and pathological variables (NPI category, tumour size, lymphocytic infiltration, grade, ER status, HER2 status, histological subtype and lymph node status) were taken as predictors of each IntClust. The predicted probability of a tumour belonging to each of the ten clusters was derived based on these models. Final IntClust subtyping was based on the highest assigned probability and these assignments compared to gold-standard subtyping based on genomic data. Also, to evaluate whether grade or receptor status explains the differences in histological types within IntClusts, a log-linear model of conditional independence: [IntClust-Grade, Histology-Grade] and a more general model that also includes the association between IntClusters and Histology: [IntClust-Grade, Histology-Grade, IntClust-Histology] were fitted. The two models were compared with the Deviance test, showing that the more general model produced a better fit to the data (*p*-value < 0.01) and, therefore, the distribution of tumour grade does not explain properly the distribution of histological types in each IntClust.

### Comparison of IntClust distribution with other classifiers

IntClust classification for the cohort was compared with PAM50 and SCMGENE classification, as described by Ali et al.^9^

### Statistical analyses

Associations were studied between IntClust and histopathological parameters and analysed by Chi-square analyses using SPSS. A *p*-value of < 0.05 was considered to be statistically significant. For association of individual subtypes of histopathological parameters with IntClusts, Pearson residuals of the Chi-squared test was calculated and a threshold of + /−2.5 was deemed significant.

### Data availability statement

All primary data, deposited at the EGA (EGAS00000000083), may be downloaded by requesting the METABRIC Data Access Committee. Gene expression data, copy number data from the original METABRIC publication can be found on the freely available cBioPortal. Published IDs for the cases included in the study have been provided in a supplementary file [Supplementary File 5: Published IDs.doc].

### Disclosure

Preliminary data related to this work was presented at 26th European Congress of Pathology, London, 2014 [Joint meeting of the European Society of Pathology and the Pathological Society of GB and Ireland].

## Electronic supplementary material

Supplementary File 1

Supplementary File 2

Supplementary File 3

Supplementary File 4

Supplementary File 5
