# Supplementary material for: Associations between genomic stratification of breast cancer and centrally reviewed tumour pathology in the METABRIC cohort
Source: NPJ Breast Cancer. 2018 Mar 7;4:5. doi: 10.1038/s41523-018-0056-8 (PMC5841292; doi:10.1038/s41523-018-0056-8)
Supplement: Supplementary file 1 [file 41523_2018_56_MOESM1_ESM.docx]

**Supplementary Files:**

**Text Summary:**

Supplementary File 1 (.doc) includes supplementary Tables including legends elaborating on patient characteristics and correlations of IntClusters with pathological grade components and PAM50/SCMGENE subtypes. It also includes analysis of deviance for models testing for conditional independence between IntClusts and histological types. A table summarising all molecular/pathological data is provided. Immuno-histochemical staining parameters are also elaborated.

Supplementary File 2 [.tif] includes figures (Suppl Fig 1a and 1b) to show immunohistochemical expression of ER/PR and HER2 within each IntClust.

Supplementary File 3 [.tif] includes figure (Suppl Fig 2) to show immunohistochemical expression of ER/PR/HER2 within each IntClust.

Supplementary File 4 [.tif] includes figures (Suppl Fig 3a and 3b) to portray the probability of prediction of IntClusters from clinicopathologial variables.

Supplementary File 5 (.doc) includes published IDs of cases in the study.

**Supplementary Table Legends**

**Suppl Table A:** Patients’ parameters of the METABRIC series (n=1643, where central pathology review with integrated genomic data was available)

**Suppl Table B:** Distribution of the components of grade: i) tubule score ii) mitoses score and iii) pleomorphism score among (a) IntClusts and (b) PAM50 subtypes in the METABRIC series [% in brackets indicates the % of cases for a particular score 1/2/3 distributed in the IntClust/PAM50 subclass]

**Suppl Table C:** Tests for conditional independence between IntClusts and histological types (HT) for the parameters: a) Grade and b) ERHER2. Analysis of deviance tables comparing deviances of Model 2 (including) and Model 1 (excluding) IntClusts and HT interactions. The highly significant p value indicates that Model 2 is the preferred model. Df=degrees of freedom; Pr (>Chi) = probability of observing Chi square statistic

**Suppl Table D:** Distribution of i) PAM50 and ii) SCMGENE types among IntClusts in the series [% in brackets indicate the % of cases in the IntClust belonging to PAM50/SCMGENE gene subclass]

**Suppl Table E:** Summary characteristics of IntClusts generated through the METABRIC analysis (strongest histological associations indicated with *)

**Suppl Table F:** Antibody panel used for immunohistochemical profiling. CC1 [Ventana Antigen Retrieval Cell Conditioning Buffer 1 (Tris/Borate/EDTA Buffer pH8.0); H [Human]; Rb [Rabbit]; Ms [Mouse]; DAB [DAB (3,3'-diaminobenzidine); HRP [Horse-radish peroxidase]; CISH [chromogenic in-situ hybridisation]

**Supplementary Table A: Patient Parameters**

| **Parameter** | **Descriptions of Parameters** | | | | |
| --- | --- | --- | --- | --- | --- |
| Age | **Mean** 61.25 years | **Range** 22-96 years |  |  |  |
| Menopausal status | **Pre** 364 (22.2%) | **Post** 1268 (77.2%) | **Unavailable** 11 (0.7%) |  |  |
| T Stage | **T1** 708 (43%) | **T2** 785 (48%) | **T3** 122 (7%) | **T4** 10 (1%) | **Unavailable** 18 (1%) |
| Lymph Node status | **Positive**  868 (52.8%) | **Negative**  770 (46.9%) | **Unavailable**  5 (0.3%) |  |  |
| ER status | **Positive** 1261 (76.7%) | **Negative** 374  (22.8%) | **Unavailable** 8  (0.5%) |  |  |
| PR status | **Positive** 675 (41%) | **Negative** 657 (40%) | **Unavailable** 311  (19%) |  |  |
| Her2 status | **Positive** 198 (12.1%) | **Negative** 1413  (86%) | **Equivocal** 2  (0.1%) | **Unavailable** 30  (1.8%) |  |

**Supplementary Table Bi.a:** **Distribution of the tubule score among IntClusts**

| **IntCluster** | **T1** | **T2** | **T3** |
| --- | --- | --- | --- |
| **1** | 0 (0%) | 14 (4%) | 105 (9%) |
| **2** | 0 (0%) | 10 (3%) | 55 (5%) |
| **3** | 16 (30%) | 76 (21%) | 148 (12%) |
| **4** | 11 (20%) | 57 (16%) | 211 (17%) |
| **5** | 0 (0%) | 20 (6%) | 129 (11%) |
| **6** | 1 (2%) | 11 (3%) | 57 (5%) |
| **7** | 5 (9%) | 53 (15%) | 113 (9%) |
| **8** | 19 (35%) | 98 (28%) | 125 (10%) |
| **9** | 2 (4%) | 9 (3%) | 107 (9%) |
| **10** | 0 (0%) | 8 (2%) | 176 (14%) |

**Supplementary Table Bi.b: Distribution of the tubule score among PAM50 subtypes**

| **PAM50 subtype** | **T1** | **T2** | **T3** |
| --- | --- | --- | --- |
| **Basal** | 0 (0%) | 13 (4%) | 243 (20%) |
| **Her2** | 1 (2%) | 19 (5%) | 168 (14%) |
| **LUMA** | 45 (83%) | 214 (60%) | 366 (30%) |
| **LUMB** | 3 (6%) | 69 (19%) | 334 (27%) |
| **Normal-like** | 5 (9%) | 39 (11%) | 112 (9%) |
| **Not-coded** | 0 (0%) | 2 (1%) | 3 (0.2%) |

**Supplementary Table Bii.a: Distribution of the mitotic score among IntClusts**

| **IntCluster** | **M1** | **M2** | **M3** |
| --- | --- | --- | --- |
| **1** | 57 (6%) | 32 (8%) | 30 (12%) |
| **2** | 36 (4%) | 24 (6%) | 5 (2%) |
| **3** | 201 (20%) | 34 (9%) | 5 (2%) |
| **4** | 193 (19%) | 62 (16%) | 23 (9%) |
| **5** | 62 (6%) | 49 (13%) | 38 (15%) |
| **6** | 37 (4%) | 19 (5%) | 13 (5%) |
| **7** | 137 (14%) | 28 (7%) | 6 (2%) |
| **8** | 184 (18%) | 52 (14%) | 6 (2%) |
| **9** | 61 (6%) | 34 (9%) | 23 (9%) |
| **10** | 37(4%) | 44 (12%) | 103 (41%) |

**Supplementary Table Bii.b:** **Distribution of the mitotic score among PAM50 subtypes**

| **PAM50 subtype** | **M1** | **M2** | **M3** |
| --- | --- | --- | --- |
| **Basal** | 62 (6%) | 72 (19%) | 122 (48%) |
| **Her2** | 92 (9%) | 55 (15%) | 41 (16%) |
| **LUMA** | 522 (52%) | 86 (23%) | 17 (7%) |
| **LUMB** | 206 (21%) | 138 (37%) | 62 (25%) |
| **Normal-like** | 119 (12%) | 26 (7%) | 10 (4%) |
| **Not-coded** | 4 (0%) | 1 (0%) | 0 (0%) |

**Supplementary Table Biii.a:** **Distribution of the pleomorphism score among IntClusts**

| **IntCluster** | **P1** | **P2** | **P3** |
| --- | --- | --- | --- |
| **1** | 1 (6%) | 26 (4%) | 92 (9%) |
| **2** | 0 (0%) | 25 (4%) | 40 (4%) |
| **3** | 2 (12%) | 145 (24%) | 93 (9%) |
| **4** | 9 (53%) | 110 (18%) | 160 (16%) |
| **5** | 0 (0%) | 21 (4%) | 128 (13%) |
| **6** | 0 (0%) | 21 (4%) | 48 (5%) |
| **7** | 3 (18%) | 86 (14%) | 82 (8%) |
| **8** | 1 (6%) | 127 (21%) | 114 (11%) |
| **9** | 1 (6%) | 27 (5%) | 90 (9%) |
| **10** | 0 (0%) | 10 (2%) | 174 (17%) |

**Supplementary Table Biii.b:** **Distribution of the pleomorphism score among PAM50 subtypes**

| **PAM50 subtype** | **P1** | **P2** | **P3** |
| --- | --- | --- | --- |
| **Basal** | 1 (6%) | 17 (3%) | 238 (23%) |
| **Her2** | 0 (0%) | 36 (6%) | 152 (15%) |
| **LUMA** | 9 (53%) | 337 (56%) | 279 (27%) |
| **LUMB** | 2 (12%) | 127 (21%) | 277 (27%) |
| **Normal-like** | 5 (30%) | 77 (13%) | 74 (7%) |
| **Not-coded** | 0 (0%) | 4 (1%) | 1 (0.1%) |

**Supplementary Table Ci: Analysis of deviance for models related to Grade**

| **Model number and**  **[Parameters Included]** | **Residual** | **Df Residual** | **Deviance** | **Df Deviance** | **Pr (>Chi)** |
| --- | --- | --- | --- | --- | --- |
| 1  [Freq ~ IntClust + HT + GRADE + IntClust:GRADE + HT:GRADE] | 162 | 206.22 |  |  |  |
| 2  [Freq ~ IntClust + HT + GRADE + IntClust:GRADE + HT:GRADE + IntClust:HT] | 108 | 62.50 | 54 | 143.72 | 4.413e-10 *** |

**Supplementary Table Cii**: **Analysis of deviance for models related to ERHER2**

| **Model number and**  **[Parameters Included]** | **Residual** | **Df Residual** | **Deviance** | **Df Deviance** | **Pr (>Chi)** |
| --- | --- | --- | --- | --- | --- |
| 1  [Freq ~ IntClust + HT + ERHER2 + IntClust:ERHER2 + HT:ERHER2] | 216 | 189.630 |  |  |  |
| 2  [Freq ~ IntClust + HT + ERHER2 + IntClust:ERHER2 + HT:ERHER2 + IntClust:HT] | 162 | 43.973 | 54 | 145.66 | 2.356e-10 *** |

**Supplementary Table Di: Distribution of PAM50 types among IntClusts**

| **PAM50 subtype** | **IntClust** | | | | | | | | | | **Total** |
| --- | --- | --- | --- | --- | --- | --- | --- | --- | --- | --- | --- |
|  | **1** | **2** | **3** | **4** | **5** | **6** | **7** | **8** | **9** | **10** |  |
| **LumA** | 9  (8%) | 23  (35%) | 165  (69%) | 94  (34%) | 16  (11%) | 20  (29%) | 113  (66%) | 162  (67%) | 23  (19%) | 1  (0.5%) | 626 |
| **LumB** | 79  (66%) | 31  (48%) | 35  (15%) | 25  (9%) | 30  (20%) | 34  (49%) | 36  (21%) | 68  (28%) | 57  (48%) | 13  (7%) | 408 |
| **Her2** | 17  (14%) | 6  (9%) | 7  (3%) | 30  (11%) | 83  (55%) | 8  (12%) | 9  (5%) | 5  (2%) | 17  (14%) | 8  (4%) | 190 |
| **Basal** | 7  (6%) | 2  (3%) | 2  (1%) | 47  (17%) | 14  (9%) | 1  (1%) | 3  (2%) | 1  (0.4%) | 19  (16%) | 162  (88%) | 258 |
| **Normal-like** | 7  (6%) | 3  (5%) | 28  (12%) | 83  (30%) | 8  (5%) | 6  (9%) | 9  (5%) | 7  (3%) | 4  (3%) | 1  (0.5%) | 156 |
| **Not Coded** | 0  (0%) | 0  (0%) | 3  (1%) | 1  (0.4%) | 0  (0%) | 0  (0%) | 1  (1%) | 0  (0%) | 0  (0%) | 0  (0%) | 5 |

**Supplementary Table Dii: Distribution of SCMGENE types among IntClusts**

| **SCMGENE subtype** | **IntClust** | | | | | | | | | | **Total** |
| --- | --- | --- | --- | --- | --- | --- | --- | --- | --- | --- | --- |
|  | **1** | **2** | **3** | **4** | **5** | **6** | **7** | **8** | **9** | **10** |  |
| **ER+/Her2-ve low proliferation** | 9  (8%) | 13  (20%) | 168  (70%) | 127  (45%) | 0  (0%) | 12  (17%) | 79  (46%) | 123  (51%) | 6  (5%) | 0  (0%) | 537 |
| **ER+/Her2-ve high proliferation** | 75  (63%) | 43  (66%) | 45  (19%) | 34  (12%) | 10  (7%) | 49  (71%) | 69  (40%) | 92  (38%) | 80  (67%) | 14  (8%) | 511 |
| **Her2+** | 8  (7%) | 2  (3%) | 0  (0%) | 11  (4%) | 126  (83%) | 1  (1%) | 0  (0%) | 0  (0%) | 9  (8%) | 1  (1%) | 158 |
| **ER-/HER2-** | 7  (6%) | 2  (3%) | 3  (1%) | 75  (27%) | 3  (2%) | 0  (0%) | 2  (1%) | 2  (1%) | 16  (13%) | 138  (75%) | 248 |
| **Not coded** | 20  (17%) | 5  (8%) | 24  (10%) | 33  (12%) | 12  (8%) | 7  (10%) | 21  (12%) | 26  (11%) | 9  (8%) | 32  (17%) | 189 |

**Supplementary Table E: Summary characteristics of IntClusts**

| **IntClust** | **Prognosis** | **Copy Number**  **Defects** | **Drivers** | **Histological subtype** | **Lymphocytic**  **Infiltrate** | **Receptor**  **Status**  **[IHC]** | **Major PAM50 subtype** |
| --- | --- | --- | --- | --- | --- | --- | --- |
| 1 | Intermediate | 17q23 amplification | *RPS6KB1* and *PPM1D* and have the highest prevalence of *GATA3* mutations | NST | Low | ER+, PR+, HER2-;  ER+, PR-, HER2-; | Luminal B |
| 2 | Poor | Amplification of 11q13/14 | *CCND1* (11q13.3), *EMSY* (11q13.5), *PAK1* (11q14.1) and *RSF1* (11q14.1) | NST  Lobular | Low | ER+, PR+, HER2- | Luminal B |
| 3 | Good | Very few | highest frequency of *PIK3CA*, *CDH1* and *RUNX1* mutations | NST  Mixed NST*  Tubular*  Lobular* | Low | ER+, PR+, HER2-* | Luminal A |
| 4 | Good | Very few; 20% have deletions at the T‐cell receptor (TCR) loci on chromosomes 7 (*TRG*) and 14 (*TRA*) | activation of immune pathways | NST  Tubular  Lobular  Medullary like carcinoma | High | ER+, PR+, HER2- | Luminal A |
| 5 | Extremely poor | 17q12  (ERBB2 amplification) | HER2 amplification and p53 mutations | NST* | Low | ER-PR-HER2+*; ER+PR-  HER2+* | HER2 |
| 6 | Intermediate | 8p12 amplification | ZNF703, harbours the lowest levels of *PIK3CA* mutations | NST | Low | ER+, PR+, HER2-;  ER+, PR-, HER2-*; | Luminal B |
| 7 | Good | 16p gain and 16q loss; 8q amplification; lacks 1q alteration | highest frequency of *MAP3K1* and *CTCF* mutations | NST  Lobular | Low | ER+, PR+, HER2-* | Luminal A |
| 8 | Good | 1q gain, 16q loss | *PIK3CA* and *GATA3* and *MAP2K4* mutations | NST  Mixed NST*  Lobular | Low | ER+, PR+, HER2-* | Luminal A |
| 9 | Intermediate | 8 q gain,  20q amplification | highest level of TP53 mutations among the ER‐positive subtypes; deletions of *PPP2R2A* | NST | Low | ER+, PR+, HER2- | Luminal B |
| 10 | Extremely poor in the first 5 years; better thereafter | 5q loss and 8q, 10p and12p gain | impaired DNA damage repair and cell-cycle checkpoint regulation; highest rates of TP53 mutations | NST  Medullary like carcinoma* | High | ER-PR-HER2-* | Basal |

**Supplementary Table F: Summary of immunohistochemical profiling**

| **Antigen** | **Clone** | **Source** | **Primary antibody species** | **Reactive species** | **Primary antibody dilution** | **Antigen retrieval** | **System** | **Secondary antibody** | **Detection system** | **Positive Score** |
| --- | --- | --- | --- | --- | --- | --- | --- | --- | --- | --- |
| **ER** | SP1 | Thermo-scientific | Rabbit | H, Ms | 1:50 | 1 hour, no heat;  CC1 standard | Ventana Discovery XT | Ultramap anti-Rb HRP | Ultramap DAB | ≥2 Allred |
| **PR** | PgR  636 | Dako | Mouse | H | 1:50 | 1 hour, no heat;  CC1 standard | Leica-Bond Max | Ultramap anti-Ms HRP | Ultramap DAB | ≥2 Allred |
| **HER2** | 4B5 | Ventana | Rabbit | H | Neat | 32 minutes with heat;  CC1 standard | Ventana Discovery XT | Ventana Universal | Ultramap DAB | 3+ |
| **HER2** |  | Ventana |  |  | Ready to use kit |  | Ventana (CISH) |  |  | >6.0  copies deemed as amplified |

**Supplementary Figure Legends:**

**Supplementary Fig 1** Immunohistochemical expression of **a)** ER/PR and **b)** HER2 within Integrative Clusters (IntClust) [data labels show absolute values; areas within the tiles in the spine-plot are proportional representations; X-axis: (of the whole cohort); Y axis: (within each IntClust)]

**Supplementary Fig 2** Immunohistochemical expression of ER/PR/HER2 within Integrative Clusters (IntClust) [data labels show absolute values; areas within the tiles in the spine-plot are proportional representations; X-axis: (of the whole cohort); Y axis: (within each IntClust)]

**Supplementary Fig 3a)** Agreement between IntClusts predicted from regression models based on clinicopathological variables *vis a vis* DNA/RNA classifier **3b)** Probability of prediction of individual IntClust based on clinico-pathological variables
