# Supplementary figures and images for: Associations between genomic stratification of breast cancer and centrally reviewed tumour pathology in the METABRIC cohort
Source: NPJ Breast Cancer. 2018 Mar 7;4:5. doi: 10.1038/s41523-018-0056-8 (PMC5841292; doi:10.1038/s41523-018-0056-8)

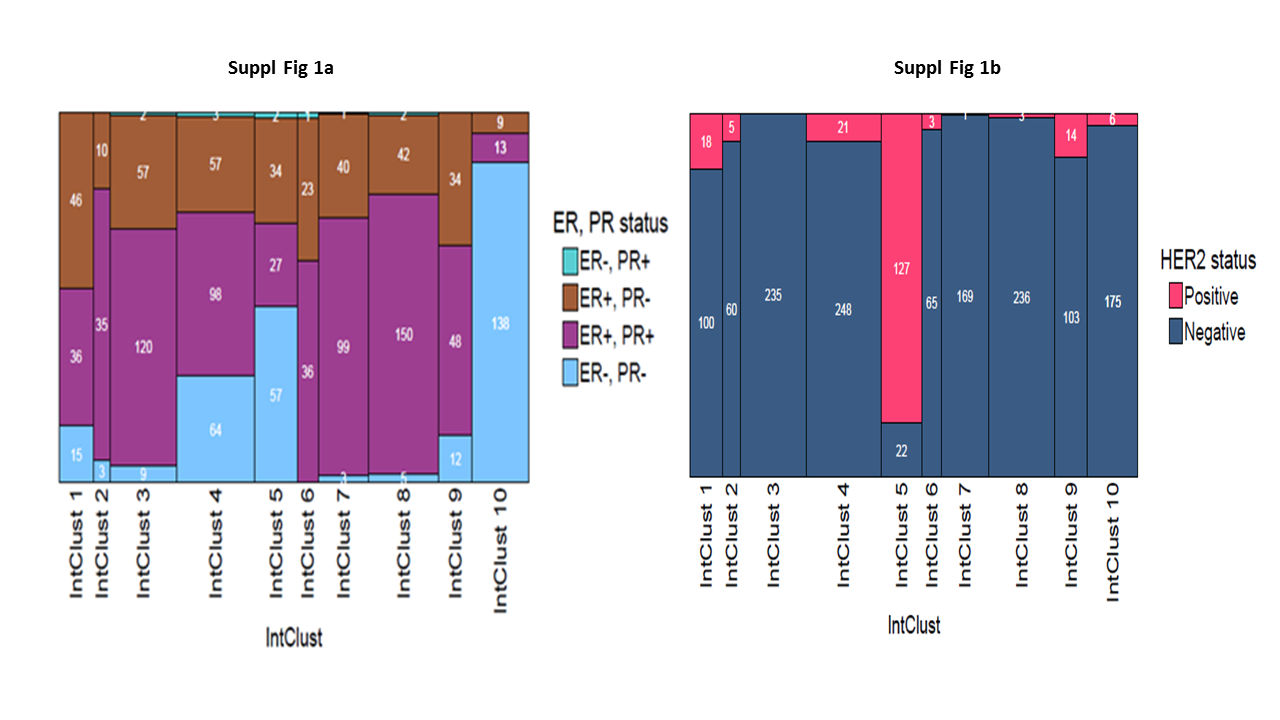

Supplement: Supplementary file 2 [file 41523_2018_56_MOESM2_ESM.tif]

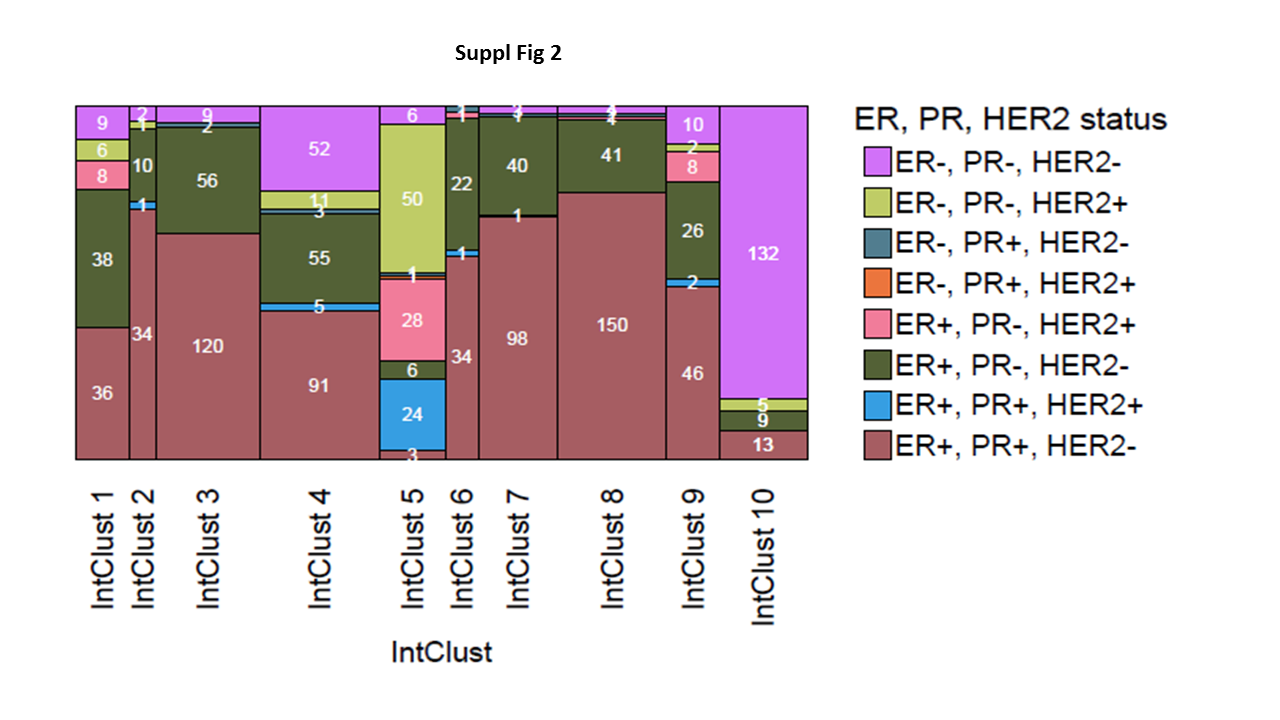

Supplement: Supplementary file 3 [file 41523_2018_56_MOESM3_ESM.tif]

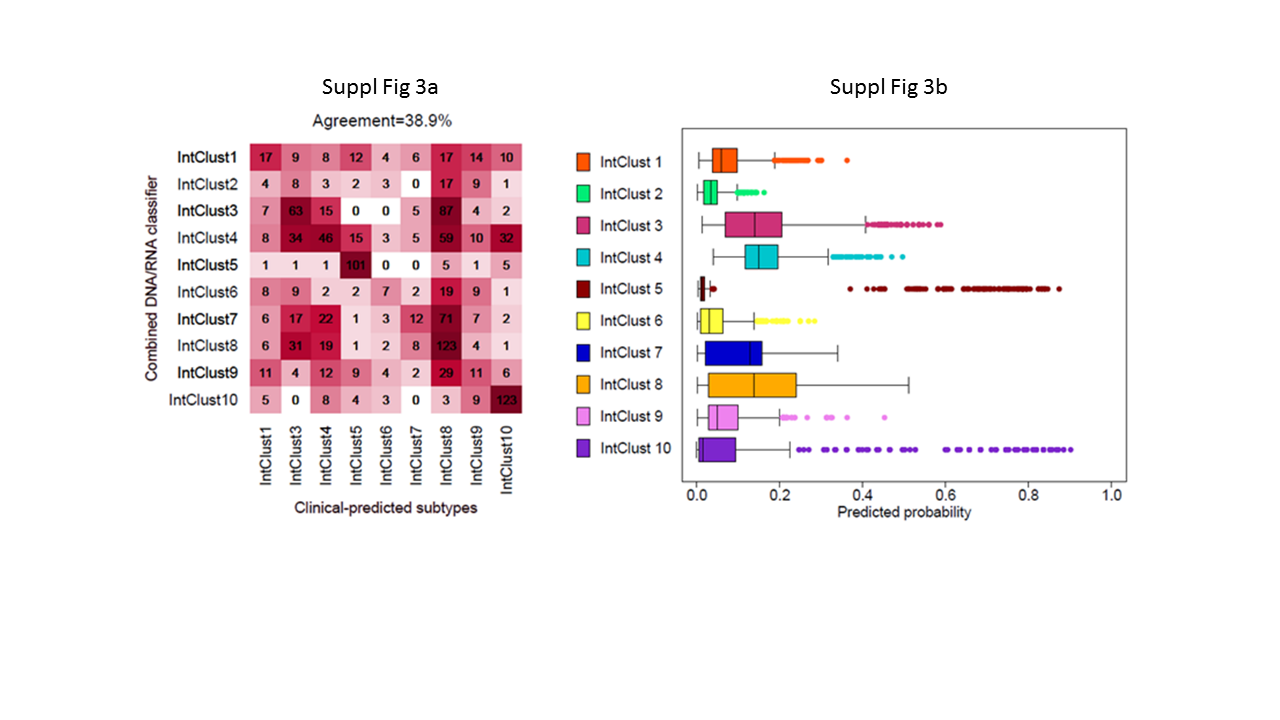

Supplement: Supplementary file 4 [file 41523_2018_56_MOESM4_ESM.tif]
