# Supplementary material for: Associations between genomic stratification of breast cancer and centrally reviewed tumour pathology in the METABRIC cohort
Source: NPJ Breast Cancer. 2018 Mar 7;4:5. doi: 10.1038/s41523-018-0056-8 (PMC5841292; doi:10.1038/s41523-018-0056-8)
Supplement: Supplementary file 5 [file 41523_2018_56_MOESM5_ESM.docx]

**Supplementary Information**

**Published IDs included in the current study**

MB-0000

MB-0002

MB-0005

MB-0006

MB-0008

MB-0010

MB-0014

MB-0020

MB-0022

MB-0028

MB-0035

MB-0036

MB-0045

MB-0046

MB-0048

MB-0050

MB-0053

MB-0054

MB-0056

MB-0059

MB-0060

MB-0062

MB-0064

MB-0066

MB-0068

MB-0079

MB-0081

MB-0083

MB-0093

MB-0095

MB-0097

MB-0099

MB-0100

MB-0101

MB-0102

MB-0106

MB-0107

MB-0108

MB-0109

MB-0111

MB-0112

MB-0113

MB-0114

MB-0115

MB-0116

MB-0117

MB-0119

MB-0120

MB-0121

MB-0122

MB-0123

MB-0124

MB-0125

MB-0126

MB-0127

MB-0128

MB-0129

MB-0130

MB-0131

MB-0133

MB-0134

MB-0135

MB-0136

MB-0138

MB-0139

MB-0140

MB-0142

MB-0143

MB-0144

MB-0145

MB-0146

MB-0147

MB-0148

MB-0149

MB-0150

MB-0151

MB-0152

MB-0154

MB-0157

MB-0158

MB-0162

MB-0163

MB-0164

MB-0165

MB-0166

MB-0167

MB-0168

MB-0169

MB-0170

MB-0171

MB-0173

MB-0174

MB-0175

MB-0176

MB-0177

MB-0178

MB-0179

MB-0180

MB-0181

MB-0184

MB-0185

MB-0188

MB-0189

MB-0191

MB-0192

MB-0193

MB-0194

MB-0195

MB-0197

MB-0198

MB-0199

MB-0200

MB-0201

MB-0202

MB-0203

MB-0204

MB-0205

MB-0206

MB-0207

MB-0211

MB-0214

MB-0215

MB-0218

MB-0220

MB-0221

MB-0222

MB-0223

MB-0224

MB-0225

MB-0226

MB-0227

MB-0228

MB-0229

MB-0231

MB-0232

MB-0233

MB-0234

MB-0235

MB-0236

MB-0238

MB-0239

MB-0241

MB-0242

MB-0243

MB-0244

MB-0245

MB-0247

MB-0248

MB-0249

MB-0253

MB-0256

MB-0257

MB-0258

MB-0259

MB-0260

MB-0261

MB-0262

MB-0263

MB-0264

MB-0265

MB-0266

MB-0268

MB-0269

MB-0270

MB-0272

MB-0273

MB-0278

MB-0279

MB-0280

MB-0282

MB-0283

MB-0285

MB-0286

MB-0287

MB-0288

MB-0289

MB-0290

MB-0291

MB-0292

MB-0293

MB-0294

MB-0295

MB-0301

MB-0302

MB-0303

MB-0304

MB-0305

MB-0306

MB-0307

MB-0308

MB-0309

MB-0310

MB-0311

MB-0312

MB-0313

MB-0314

MB-0315

MB-0316

MB-0317

MB-0318

MB-0319

MB-0320

MB-0321

MB-0322

MB-0324

MB-0325

MB-0333

MB-0336

MB-0339

MB-0340

MB-0342

MB-0343

MB-0344

MB-0345

MB-0346

MB-0347

MB-0348

MB-0349

MB-0350

MB-0351

MB-0352

MB-0353

MB-0354

MB-0356

MB-0358

MB-0359

MB-0360

MB-0361

MB-0362

MB-0363

MB-0364

MB-0365

MB-0366

MB-0367

MB-0368

MB-0369

MB-0370

MB-0371

MB-0372

MB-0373

MB-0374

MB-0375

MB-0377

MB-0378

MB-0379

MB-0380

MB-0381

MB-0382

MB-0383

MB-0384

MB-0385

MB-0386

MB-0388

MB-0389

MB-0390

MB-0391

MB-0392

MB-0393

MB-0394

MB-0395

MB-0396

MB-0397

MB-0398

MB-0399

MB-0400

MB-0401

MB-0402

MB-0404

MB-0405

MB-0406

MB-0408

MB-0410

MB-0411

MB-0412

MB-0413

MB-0414

MB-0417

MB-0418

MB-0419

MB-0420

MB-0421

MB-0422

MB-0423

MB-0425

MB-0426

MB-0427

MB-0428

MB-0429

MB-0431

MB-0432

MB-0434

MB-0435

MB-0436

MB-0437

MB-0438

MB-0439

MB-0440

MB-0442

MB-0443

MB-0444

MB-0445

MB-0446

MB-0448

MB-0449

MB-0451

MB-0452

MB-0453

MB-0454

MB-0455

MB-0458

MB-0459

MB-0460

MB-0462

MB-0463

MB-0464

MB-0465

MB-0467

MB-0468

MB-0469

MB-0470

MB-0471

MB-0474

MB-0475

MB-0476

MB-0478

MB-0479

MB-0480

MB-0481

MB-0482

MB-0483

MB-0484

MB-0485

MB-0486

MB-0487

MB-0488

MB-0489

MB-0490

MB-0491

MB-0492

MB-0494

MB-0495

MB-0496

MB-0500

MB-0501

MB-0502

MB-0503

MB-0504

MB-0505

MB-0506

MB-0507

MB-0508

MB-0510

MB-0511

MB-0512

MB-0513

MB-0516

MB-0519

MB-0521

MB-0524

MB-0525

MB-0526

MB-0527

MB-0528

MB-0529

MB-0531

MB-0532

MB-0534

MB-0535

MB-0536

MB-0537

MB-0538

MB-0540

MB-0541

MB-0542

MB-0543

MB-0544

MB-0545

MB-0546

MB-0549

MB-0550

MB-0551

MB-0552

MB-0553

MB-0554

MB-0558

MB-0559

MB-0564

MB-0568

MB-0569

MB-0570

MB-0571

MB-0573

MB-0574

MB-0575

MB-0576

MB-0577

MB-0578

MB-0579

MB-0580

MB-0581

MB-0583

MB-0584

MB-0585

MB-0586

MB-0587

MB-0588

MB-0589

MB-0590

MB-0591

MB-0593

MB-0594

MB-0596

MB-0597

MB-0598

MB-0599

MB-0600

MB-0601

MB-0603

MB-0605

MB-0606

MB-0607

MB-0608

MB-0609

MB-0610

MB-0611

MB-0613

MB-0614

MB-0616

MB-0617

MB-0618

MB-0619

MB-0620

MB-0621

MB-0623

MB-0624

MB-0626

MB-0627

MB-0628

MB-0630

MB-0631

MB-0632

MB-0634

MB-0635

MB-0636

MB-0637

MB-0638

MB-0639

MB-0640

MB-0641

MB-0642

MB-0643

MB-0644

MB-0646

MB-0649

MB-0650

MB-0652

MB-0653

MB-0654

MB-0655

MB-0656

MB-0657

MB-0658

MB-0659

MB-0660

MB-0661

MB-0662

MB-0664

MB-0666

MB-0667

MB-0869

MB-0872

MB-0874

MB-0877

MB-0880

MB-0882

MB-0884

MB-0891

MB-0893

MB-0895

MB-0897

MB-0899

MB-0901

MB-0904

MB-0906

MB-6006

MB-6007

MB-6008

MB-6010

MB-6011

MB-6012

MB-6014

MB-6016

MB-6018

MB-6020

MB-6021

MB-6022

MB-6023

MB-6026

MB-6029

MB-6030

MB-6036

MB-6039

MB-6042

MB-6044

MB-6047

MB-6048

MB-6049

MB-6050

MB-6051

MB-6052

MB-6053

MB-6055

MB-6058

MB-6059

MB-6060

MB-6062

MB-6063

MB-6068

MB-6069

MB-6071

MB-6075

MB-6077

MB-6079

MB-6080

MB-6082

MB-6083

MB-6085

MB-6092

MB-6097

MB-6098

MB-6100

MB-6101

MB-6105

MB-6107

MB-6108

MB-6113

MB-6114

MB-6116

MB-6118

MB-6122

MB-6124

MB-6125

MB-6131

MB-6133

MB-6135

MB-6138

MB-6141

MB-6143

MB-6144

MB-6145

MB-6146

MB-6147

MB-6149

MB-6150

MB-6152

MB-6154

MB-6156

MB-6157

MB-6160

MB-6163

MB-6164

MB-6167

MB-6168

MB-6169

MB-6171

MB-6178

MB-6179

MB-6181

MB-6182

MB-6183

MB-6184

MB-6185

MB-6188

MB-6189

MB-6190

MB-6192

MB-6194

MB-6195

MB-6200

MB-6201

MB-6204

MB-6207

MB-6208

MB-6211

MB-6212

MB-6213

MB-6214

MB-6217

MB-6218

MB-6223

MB-6224

MB-6225

MB-6226

MB-6228

MB-6229

MB-6230

MB-6231

MB-6232

MB-6233

MB-6234

MB-6237

MB-6238

MB-6239

MB-6242

MB-6245

MB-6246

MB-6248

MB-6251

MB-6253

MB-6254

MB-6256

MB-6257

MB-6263

MB-6271

MB-6272

MB-6273

MB-6280

MB-6281

MB-6283

MB-6284

MB-6286

MB-6287

MB-6288

MB-6297

MB-6300

MB-6302

MB-6305

MB-6306

MB-6308

MB-6312

MB-6314

MB-6317

MB-6318

MB-6319

MB-6322

MB-6327

MB-6328

MB-6329

MB-6330

MB-6334

MB-6336

MB-6337

MB-6344

MB-6346

MB-6359

MB-6363

MB-2513

MB-2517

MB-2536

MB-2556

MB-2564

MB-2610

MB-2613

MB-2614

MB-2616

MB-2617

MB-2618

MB-2624

MB-2626

MB-2629

MB-2632

MB-2634

MB-2642

MB-2643

MB-2645

MB-2669

MB-2686

MB-2705

MB-2708

MB-2711

MB-2712

MB-2718

MB-2721

MB-2724

MB-2725

MB-2728

MB-2730

MB-2735

MB-2742

MB-2744

MB-2745

MB-2747

MB-2749

MB-2750

MB-2752

MB-2753

MB-2754

MB-2758

MB-2760

MB-2763

MB-2764

MB-2765

MB-2767

MB-2769

MB-2770

MB-2771

MB-2772

MB-2774

MB-2778

MB-2779

MB-2781

MB-2786

MB-2790

MB-2791

MB-2792

MB-2793

MB-2795

MB-2796

MB-2797

MB-2801

MB-2803

MB-2814

MB-2815

MB-2819

MB-2820

MB-2821

MB-2823

MB-2827

MB-2833

MB-2834

MB-2835

MB-2838

MB-2840

MB-2842

MB-2843

MB-2844

MB-2845

MB-2846

MB-2847

MB-2848

MB-2849

MB-2850

MB-2851

MB-2853

MB-2854

MB-2857

MB-2858

MB-2863

MB-2867

MB-2895

MB-2896

MB-2900

MB-2901

MB-2904

MB-2912

MB-2916

MB-2917

MB-2919

MB-2922

MB-2923

MB-2927

MB-2929

MB-2931

MB-2932

MB-2933

MB-2939

MB-2944

MB-2947

MB-2951

MB-2952

MB-2953

MB-2954

MB-2957

MB-2960

MB-2963

MB-2964

MB-2966

MB-2969

MB-2970

MB-2971

MB-2977

MB-2983

MB-2984

MB-2990

MB-2993

MB-2994

MB-2996

MB-2999

MB-3001

MB-3002

MB-3005

MB-3006

MB-3007

MB-3008

MB-3013

MB-3014

MB-3016

MB-3021

MB-3025

MB-3026

MB-3028

MB-3031

MB-3032

MB-3033

MB-3035

MB-3037

MB-3046

MB-3049

MB-3050

MB-3057

MB-3058

MB-3060

MB-3062

MB-3063

MB-3064

MB-3067

MB-3079

MB-3083

MB-3085

MB-3088

MB-3092

MB-3102

MB-3103

MB-3104

MB-3105

MB-3110

MB-3121

MB-3122

MB-3123

MB-3153

MB-3165

MB-3167

MB-3171

MB-3181

MB-3211

MB-3218

MB-3222

MB-3228

MB-3235

MB-3252

MB-3253

MB-3254

MB-3266

MB-3271

MB-3272

MB-3275

MB-3277

MB-3292

MB-3295

MB-3297

MB-3298

MB-3300

MB-3301

MB-3303

MB-3328

MB-3329

MB-3341

MB-3344

MB-3350

MB-3351

MB-3355

MB-3357

MB-3360

MB-3361

MB-3363

MB-3365

MB-3367

MB-3371

MB-3378

MB-3379

MB-3381

MB-3382

MB-3383

MB-3386

MB-3388

MB-3389

MB-3395

MB-3396

MB-3402

MB-3403

MB-3412

MB-3417

MB-3429

MB-3430

MB-3435

MB-3436

MB-3437

MB-3439

MB-3450

MB-3452

MB-3453

MB-3459

MB-3462

MB-3466

MB-3467

MB-3470

MB-3476

MB-3479

MB-3487

MB-3488

MB-3490

MB-3492

MB-3497

MB-3500

MB-3502

MB-3506

MB-3510

MB-3525

MB-3526

MB-3528

MB-3530

MB-3536

MB-3545

MB-3547

MB-3548

MB-3556

MB-3567

MB-3576

MB-3582

MB-3600

MB-3606

MB-3614

MB-3702

MB-3706

MB-3707

MB-3711

MB-3748

MB-3752

MB-3754

MB-3781

MB-3797

MB-3823

MB-3824

MB-3838

MB-3840

MB-3842

MB-3850

MB-3852

MB-3854

MB-3865

MB-3866

MB-3871

MB-3874

MB-3978

MB-4000

MB-4001

MB-4003

MB-4004

MB-4005

MB-4008

MB-4010

MB-4011

MB-4012

MB-4015

MB-4017

MB-4018

MB-4024

MB-4033

MB-4046

MB-4059

MB-4079

MB-4091

MB-4098

MB-4119

MB-4120

MB-4126

MB-4127

MB-4139

MB-4140

MB-4141

MB-4145

MB-4146

MB-4148

MB-4171

MB-4173

MB-4189

MB-4212

MB-4213

MB-4222

MB-4224

MB-4230

MB-4233

MB-4234

MB-4235

MB-4236

MB-4250

MB-4254

MB-4264

MB-4266

MB-4270

MB-4274

MB-4276

MB-4278

MB-4281

MB-4282

MB-4283

MB-4289

MB-4292

MB-4293

MB-4298

MB-4300

MB-4303

MB-4306

MB-4310

MB-4313

MB-4317

MB-4318

MB-4322

MB-4323

MB-4324

MB-4328

MB-4329

MB-4331

MB-4332

MB-4333

MB-4339

MB-4341

MB-4342

MB-4343

MB-4348

MB-4350

MB-4351

MB-4353

MB-4354

MB-4357

MB-4360

MB-4368

MB-4374

MB-4375

MB-4390

MB-4395

MB-4407

MB-4408

MB-4416

MB-4417

MB-4418

MB-4426

MB-4434

MB-4442

MB-4484

MB-4529

MB-4548

MB-4557

MB-4564

MB-4578

MB-4591

MB-4593

MB-4598

MB-4599

MB-4601

MB-4602

MB-4607

MB-4616

MB-4618

MB-4621

MB-4622

MB-4623

MB-4626

MB-4627

MB-4630

MB-4633

MB-4634

MB-4639

MB-4640

MB-4641

MB-4642

MB-4643

MB-4644

MB-4648

MB-4649

MB-4651

MB-4654

MB-4655

MB-4660

MB-4661

MB-4663

MB-4665

MB-4666

MB-4667

MB-4669

MB-4670

MB-4671

MB-4672

MB-4673

MB-4674

MB-4675

MB-4679

MB-4681

MB-4682

MB-4685

MB-4686

MB-4687

MB-4688

MB-4691

MB-4694

MB-4695

MB-4696

MB-4697

MB-4698

MB-4701

MB-4702

MB-4704

MB-4705

MB-4706

MB-4707

MB-4708

MB-4709

MB-4710

MB-4711

MB-4712

MB-4714

MB-4715

MB-4716

MB-4717

MB-4718

MB-4719

MB-4721

MB-4722

MB-4723

MB-4724

MB-4725

MB-4729

MB-4730

MB-4731

MB-4732

MB-4733

MB-4735

MB-4737

MB-4738

MB-4739

MB-4741

MB-4742

MB-4743

MB-4744

MB-4745

MB-4746

MB-4749

MB-4750

MB-4752

MB-4757

MB-4758

MB-4760

MB-4762

MB-4763

MB-4764

MB-4767

MB-4769

MB-4770

MB-4771

MB-4778

MB-4779

MB-4784

MB-4785

MB-4787

MB-4790

MB-4791

MB-4792

MB-4793

MB-4794

MB-4796

MB-4797

MB-4800

MB-4801

MB-4802

MB-4805

MB-4806

MB-4814

MB-4818

MB-4820

MB-4822

MB-4825

MB-4827

MB-4828

MB-4829

MB-4832

MB-4834

MB-4836

MB-4838

MB-4839

MB-4843

MB-4845

MB-4846

MB-4849

MB-4853

MB-4855

MB-4858

MB-4859

MB-4860

MB-4862

MB-4865

MB-4866

MB-4867

MB-4869

MB-4870

MB-4871

MB-4872

MB-4873

MB-4878

MB-4879

MB-4880

MB-4881

MB-4883

MB-4886

MB-4887

MB-4888

MB-4893

MB-4894

MB-4896

MB-4898

MB-4899

MB-4900

MB-4904

MB-4906

MB-4908

MB-4911

MB-4912

MB-4925

MB-4928

MB-4929

MB-4930

MB-4931

MB-4933

MB-4934

MB-4935

MB-4937

MB-4938

MB-4941

MB-4942

MB-4944

MB-4945

MB-4949

MB-4952

MB-4956

MB-4957

MB-4959

MB-4961

MB-4962

MB-4965

MB-4966

MB-4967

MB-4968

MB-4969

MB-4970

MB-4974

MB-4976

MB-4977

MB-4978

MB-4981

MB-4982

MB-4986

MB-4991

MB-4992

MB-4993

MB-4994

MB-4996

MB-4998

MB-4999

MB-5004

MB-5008

MB-5011

MB-5013

MB-5014

MB-5015

MB-5018

MB-5019

MB-5020

MB-5022

MB-5027

MB-5033

MB-5035

MB-5039

MB-5040

MB-5041

MB-5043

MB-5044

MB-5045

MB-5048

MB-5049

MB-5050

MB-5052

MB-5053

MB-5057

MB-5058

MB-5059

MB-5060

MB-5061

MB-5062

MB-5063

MB-5064

MB-5065

MB-5066

MB-5068

MB-5070

MB-5072

MB-5074

MB-5076

MB-5078

MB-5079

MB-5081

MB-5084

MB-5086

MB-5088

MB-5093

MB-5097

MB-5098

MB-5100

MB-5101

MB-5102

MB-5104

MB-5105

MB-5106

MB-5107

MB-5109

MB-5110

MB-5113

MB-5114

MB-5115

MB-5116

MB-5117

MB-5118

MB-5119

MB-5120

MB-5121

MB-5122

MB-5123

MB-5124

MB-5126

MB-5127

MB-5130

MB-5131

MB-5134

MB-5135

MB-5136

MB-5137

MB-5138

MB-5139

MB-5140

MB-5143

MB-5144

MB-5145

MB-5147

MB-5148

MB-5152

MB-5155

MB-5157

MB-5158

MB-5160

MB-5161

MB-5163

MB-5164

MB-5166

MB-5167

MB-5169

MB-5173

MB-5174

MB-5175

MB-5177

MB-5178

MB-5179

MB-5182

MB-5183

MB-5184

MB-5185

MB-5188

MB-5189

MB-5191

MB-5193

MB-5195

MB-5196

MB-5197

MB-5199

MB-5200

MB-5201

MB-5205

MB-5206

MB-5208

MB-5209

MB-5211

MB-5213

MB-5214

MB-5215

MB-5218

MB-5221

MB-5222

MB-5223

MB-5225

MB-5226

MB-5227

MB-5228

MB-5229

MB-5230

MB-5231

MB-5232

MB-5233

MB-5235

MB-5236

MB-5238

MB-5239

MB-5240

MB-5243

MB-5251

MB-5253

MB-5255

MB-5256

MB-5258

MB-5259

MB-5260

MB-5261

MB-5264

MB-5266

MB-5267

MB-5268

MB-5270

MB-5271

MB-5272

MB-5273

MB-5275

MB-5277

MB-5278

MB-5279

MB-5281

MB-5284

MB-5287

MB-5288

MB-5289

MB-5290

MB-5291

MB-5292

MB-5293

MB-5294

MB-5295

MB-5296

MB-5298

MB-5299

MB-5300

MB-5301

MB-5302

MB-5305

MB-5306

MB-5308

MB-5310

MB-5311

MB-5312

MB-5313

MB-5315

MB-5317

MB-5318

MB-5322

MB-5323

MB-5324

MB-5325

MB-5326

MB-5327

MB-5328

MB-5329

MB-5330

MB-5331

MB-5332

MB-5334

MB-5335

MB-5338

MB-5339

MB-5341

MB-5345

MB-5346

MB-5347

MB-5348

MB-5349

MB-5350

MB-5351

MB-5358

MB-5360

MB-5361

MB-5364

MB-5365

MB-5366

MB-5368

MB-5369

MB-5370

MB-5373

MB-5377

MB-5378

MB-5381

MB-5382

MB-5383

MB-5384

MB-5385

MB-5386

MB-5387

MB-5388

MB-5389

MB-5390

MB-5393

MB-5394

MB-5395

MB-5396

MB-5397

MB-5398

MB-5399

MB-5401

MB-5402

MB-5403

MB-5404

MB-5406

MB-5408

MB-5409

MB-5410

MB-5411

MB-5412

MB-5413

MB-5414

MB-5417

MB-5418

MB-5421

MB-5422

MB-5424

MB-5425

MB-5427

MB-5428

MB-5429

MB-5431

MB-5432

MB-5433

MB-5434

MB-5440

MB-5441

MB-5442

MB-5444

MB-5446

MB-5447

MB-5450

MB-5451

MB-5452

MB-5454

MB-5455

MB-5457

MB-5459

MB-5460

MB-5463

MB-5464

MB-5465

MB-5467

MB-5468

MB-5470

MB-5471

MB-5472

MB-5473

MB-5474

MB-5477

MB-5478

MB-5481

MB-5482

MB-5483

MB-5484

MB-5485

MB-5486

MB-5489

MB-5491

MB-5492

MB-5493

MB-5495

MB-5497

MB-5498

MB-5499

MB-5502

MB-5505

MB-5510

MB-5511

MB-5513

MB-5514

MB-5518

MB-5519

MB-5520

MB-5521

MB-5525

MB-5526

MB-5527

MB-5529

MB-5530

MB-5531

MB-5532

MB-5533

MB-5534

MB-5535

MB-5540

MB-5541

MB-5543

MB-5548

MB-5549

MB-5550

MB-5551

MB-5552

MB-5553

MB-5554

MB-5556

MB-5560

MB-5562

MB-5563

MB-5565

MB-5566

MB-5567

MB-5571

MB-5572

MB-5575

MB-5576

MB-5577

MB-5579

MB-5580

MB-5582

MB-5583

MB-5584

MB-5585

MB-5588

MB-5589

MB-5590

MB-5591

MB-5592

MB-5593

MB-5596

MB-5597

MB-5599

MB-5601

MB-5602

MB-5603

MB-5604

MB-5605

MB-5613

MB-5614

MB-5616

MB-5617

MB-5620

MB-5622

MB-5623

MB-5624

MB-5625

MB-5626

MB-5628

MB-5629

MB-5632

MB-5634

MB-5635

MB-5636

MB-5638

MB-5641

MB-5642

MB-5645

MB-5646

MB-5647

MB-5648

MB-5651

MB-5653

MB-5654

MB-5656
